# Supplementary material for: Examination of an averaging method for estimating repulsion and attraction interactions in moving groups
Source: PLoS One. 2020 Dec 9;15(12):e0243631. doi: 10.1371/journal.pone.0243631 (PMC7725364; doi:10.1371/journal.pone.0243631)
Supplement: S1 File — (PDF) [file pone.0243631.s001.pdf]

# Examination of an averaging method for estimating repulsion and attraction interactions in moving groups - Supporting Information

Rajnesh K. Mudaliar, Timothy M. Schaerf

## S1 Dimensions and use of bins in determining interaction rules

### S1.1 Division of local domain into bin regions

a. **Overlapping bins for $(x, y)$ coordinates of individuals**
We partition the domain where $-L < x_{ij,relative}(t) \leq L, -L < y_{ij,relative}(t) \leq L$, centered on each focal individual $i$, into a set of overlapping square bins of side length $m$ identified by paired column and row indices $(a, b)$, such that the left edges and bottom edges of consecutive bins were seperated by $n < m$. In other words the left edges of the bins were located at $x_{a,left} = -L, -L + n, -L + 2n, ..., L - n$, the right edges of the bins were located at $x_{a,right} = -L+m, -L+m+n, -L+m+2n, ..., L$, the bottom edges of the bins were located at $y_{b,bottom} = -L, -L+n, -L+2n, ..., L-n$ and the top edges of the bins were located at $y_{b,top} = -L+m, -L+m+n, -L+m+2n, ..., L$.

b. **Speed bins**
For our analysis of data generated by the ODE model, we further subdivided our data based on the speed of the focal individual. To construct this additional layer of binning, we first determined the quartiles, particularly the lower (LQ) and upper (UQ) quartiles, and the inter-quartile range (IQR) for the speeds of all individuals across all simulated data. We then subdivided our binned data over 5 equal intervals of speed. The lower boundaries for these bins were $s_{c,left} = LQ, LQ + \frac{IQR}{5}, LQ + \frac{2\times IQR}{5}, LQ + \frac{3\times IQR}{5}, UQ - \frac{IQR}{5}$ and the upper boundaries of the same bins were $s_{c,right} = LQ + \frac{IQR}{5}, LQ + \frac{2\times IQR}{5}, LQ + \frac{3\times IQR}{5}, UQ - \frac{IQR}{5}, UQ$ respectively.

### S1.2 Allocating data into respective bins

a. **Changes in speed and direction as a function of the relative coordinates of groupmates.** For all individuals $i$, for every time step $t$, and looping over all other individuals $j \neq i$ across all data from the same set and time interval, we placed the change in direction of motion over time of focal individual $i$ given by equation (2.6) and the change in speed over time of focal individual $i$ given by equation (2.8)

into the bin with column and row indices $(a, b)$, if $x_{a,left} < x_{ij,relative}(t) \leq x_{a,right}$ and $y_{b,bottom} < y_{ij,relative}(t) \leq y_{b,top}$.

b. **Changes in speed and direction as a function of the relative coordinates of groupmates and own speed.** Applying a similar method for aggregating data to that described above, we deposited the change in direction of motion over time of focal individual $i$ given by equation (2.6) and the change in speed over time for focal individual $i$ given by equation (2.8) into the bin $(a, b, c)$, if $x_{a,left} < x_{ij,relative}(t) \leq x_{a,right}$, $y_{b,bottom} < y_{ij,relative}(t) \leq y_{b,top}$ and $s_{c,left} < s_i(t) \leq s_{c,right}$.

# S2 Three zone self-propelled individual simulation model

## S2.1 Initial conditions

In this study $N$ individuals were initially distributed randomly inside the square with $-\sqrt{2}\, r_a \leq x \leq \sqrt{2}\, r_a$ and $-\sqrt{2}\, r_a \leq y \leq \sqrt{2}\, r_a$ . We denote the position of each individual $i$ via its position vector $\mathbf{c}_i(t) = x_i(t)\mathbf{i} + y_i(t)\mathbf{j}$. The velocity of an individual at each time $t$ is defined as $\mathbf{V}_i(t) = u_i(t)\mathbf{i} + v_i(t)\mathbf{j}$ and the individual's direction (unit velocity) vector is defined as $\hat{\mathbf{V}}_i(t) = \hat{u}_i(t)\mathbf{i} + \hat{v}_i(t)\mathbf{j}$, where $\hat{u}_i(t) = u_i(t)/\sqrt{u_i^2(t) + v_i^2(t)}$ and $\hat{v}_i(t) = v_i(t)/\sqrt{u_i^2(t) + v_i^2(t)}$. The initial velocity of the individuals was assigned as $\mathbf{V}_i(0) = u_i(0)\mathbf{i} + v_i(0)\mathbf{j}$, where $u_i(0) = s\cos(\theta)$, $v_i(0) = s\sin(\theta)$, $\theta$ was a uniformly distributed random variable on $(0, 2\pi)$, and $s$ was the constant speed of all individuals.

## S2.2 Interactions

When neighbours are in a focal individual $i$'s ZOR, then the focal individual will act preferentially to avoid collisions by turning away from these neighbours. To enact turning based avoidance, each individual $i$ that has group mates in its ZOR at time $t$ prefers that its own direction of motion at time $t + \Delta t$ is the negative of the average direction to neighbouring individuals in the ZOR at time $t$, that is

$$\mathbf{d}_{r_i}(t + \Delta t) = -\sum_{j \in \text{ZOR},\, j \neq i} \frac{\mathbf{r}_{ij}(t)}{||\mathbf{r}_{ij}(t)||}. \tag{S2.1}$$

where $\mathbf{r}_{ij}(t) = \hat{x}_{ij}(t)\mathbf{i} + \hat{y}_{ij}(t)\mathbf{j}$ is the vector from the position of individual $i$ to the position of individual $j$. We denote the total number of individuals in individual $i$'s ZOR at time $t$ as $n_r$. The unit direction vector associated with an individual's preferred direction of motion based on repulsion interactions is $\hat{\mathbf{d}}_{r_i} = \frac{\mathbf{d}_{r_i}}{||\mathbf{d}_{r_i}||}$.

If there are no neighbours in individual $i$'s zone of repulsion then that individual will respond to the individuals present in its ZOO and ZOA. We denote the number of dectectable neighbours in individual $i$'s ZOO as $n_o$; such neighbours satisfy the condition that $r_r \leq ||\mathbf{r}_{ij}|| < r_o$. Similarly, we denote the number of detectable neighbours in individual

$i$'s ZOA as $n_a$; these neighbours satisfy the condition that $r_o \leq ||\mathbf{r}_{ij}|| < r_a$. The radial width of the ZOO is $\Delta r_o = r_o - r_r$ and the radial width of the ZOA is $\Delta r_a = r_a - r_o$.

Individual's prefer to align their direction of motion with that of neighbours in their ZOO. This preferred direction based on orientation interactions is given by

$$\mathbf{d}_{o_i}(t + \Delta t) = \sum_{j \in \text{ZOO}} \frac{\mathbf{V}_j(t)}{||\mathbf{V}_j(t)||}. \tag{S2.2}$$

where $||\mathbf{V}_j(t)|| = \sqrt{(u_j)^2 + (v_j)^2}$. The associated unit direction vector is $\hat{\mathbf{d}}_{o_i}v = \frac{\mathbf{d}_{o_i}(t+\Delta t)}{||\mathbf{d}_{o_i}(t+\Delta t)||}$.

Individual's will attempt to move towards neighbours in their ZOA, with their preferred direction of motion based on attraction interactions alone given by

$$\mathbf{d}_{a_i}(t + \Delta t) = \sum_{j \in \text{ZOA}} \frac{\mathbf{r}_{ij}(t)}{||\mathbf{r}_{ij}(t)||}. \tag{S2.3}$$

The associated unit direction vector for movement towards neighbours in an individuals ZOA is $\hat{\mathbf{d}}_{a_i}(t + \Delta t) = \frac{\mathbf{d}_{a_i}(t+\Delta t)}{||\mathbf{d}_{a_i}(t+\Delta t)||}$.

We consider the following five cases to determine the final intended direction $\mathbf{d}_i(t + \Delta t)$ for each individual $i$:

- Case 1: if $n_r \neq 0$ then $\mathbf{d}_i(t + \Delta t) = \hat{\mathbf{d}}_{r_i}(t + \Delta t)$. If not, go to Case 2.
- Case 2: if $n_o \neq 0$ and $n_r = 0$ and $n_a = 0$ then $\mathbf{d}_i(t + \Delta t) = \hat{\mathbf{d}}_{o_i}(t + \Delta t)$. If not, go to Case 3.
- Case 3: if $n_a \neq 0$ and $n_r = 0$ and $n_o = 0$ then $\mathbf{d}_i(t + \Delta t) = \hat{\mathbf{d}}_{a_i}(t + \Delta t)$. If not, go to Case 4.
- Case 4: if $n_o \neq 0$ and $n_a \neq 0$ and $n_r = 0$ then $\mathbf{d}_i(t+\Delta t) = \frac{1}{2}(\hat{\mathbf{d}}_{o_i}(t+\Delta t)+\hat{\mathbf{d}}_{a_i}(t+\Delta t))$. If not, go to Case 5.
- Case 5: if $n_o = 0$ and $n_a = 0$ and $n_r = 0$ then $\mathbf{d}_i(t + \Delta t) = \hat{\mathbf{V}}_i(t)$.

Then the unit final intended direction vector is calculated as $\hat{\mathbf{d}}_i(t+\Delta t) = \frac{\mathbf{d}_i(t+\Delta t)}{||\mathbf{d}_i(t+\Delta t)||} = \hat{d}_{x_i}(t+\Delta t)\mathbf{i} + \hat{d}_{y_i}(t + \Delta t)\mathbf{j}$. We add a small amount noise $\Delta\theta_\eta$ to each component of the unit final intended direction vector and define $\mathbf{d}'_i(t+\Delta t) = (\hat{d}_{x_i}(t+\Delta t)+\Delta\theta_\eta)\mathbf{i}+(\hat{d}_{y_i}(t+\Delta t)+\Delta\theta_\eta)\mathbf{j}$. $\Delta\theta_\eta$ is a normally distributed random variate with mean equal to zero, and a standard deviation of $\eta$, where $\eta$ is usually a relatively small positive number. We normalize $\mathbf{d}'_i(t+\Delta t)$ to obtain $\hat{\mathbf{d}}'_i(t + \Delta t)$.

Next we limit the turning rate of each individual by setting a maximum turning rate of $\theta_{\Delta t}$. We do this by finding the angle $\beta_i(t + \Delta t)$ between $\hat{\mathbf{V}}_i(t)$ and $\hat{\mathbf{d}}'_i(t + \Delta t)$, that is

$$\beta_i(t + \Delta t) = \arccos(\hat{\mathbf{V}}_i(t) \cdot \hat{\mathbf{d}}'_i(t + \Delta t)). \tag{S2.4}$$

If $\beta_i(t + \Delta t) \leq \theta_{\Delta t}$ then we set $\hat{\mathbf{V}}_i(t + \Delta t) = \hat{\mathbf{d}}'_i(t + \Delta t)$. If not, then we must rotate $\hat{\mathbf{V}}_i(t)$ by an angle of $\theta_{\Delta t}$ towards $\hat{\mathbf{d}}'_i(t + \Delta t))$. To do this, we apply the following steps:

i Calculate $\mathbf{a}_i(t+\Delta t)$, the component of $\hat{\mathbf{d}}_i'(t+\Delta t))$ perpendicular to $\hat{\mathbf{V}}_i(t)$ using

$$\mathbf{a}_i(t+\Delta t) = \hat{\mathbf{d}}_i'(t+\Delta t)) - ((\hat{\mathbf{V}}_i(t))(\hat{\mathbf{V}}_i(t) \cdot \hat{\mathbf{d}}_i'(t+\Delta t))). \tag{S2.5}$$

ii Determine the unit vector $\hat{\mathbf{a}}_i(t+\Delta t) = \frac{\mathbf{a}_i(t+\Delta t)}{||\mathbf{a}_i(t+\Delta t)||}$.

iii Calculate the new direction using

$$\hat{\mathbf{V}}_i(t+\Delta t) = \hat{\mathbf{V}}_i(t)\cos(\theta_{\Delta t}) + \hat{\mathbf{a}}_i(t+\Delta t)\sin(\theta_{\Delta t}). \tag{S2.6}$$

Finally we compute the position of the individual at the next time step via:

$$\mathbf{c}_i(t+\Delta t) = \mathbf{c}_i(t) + s\Delta t\hat{\mathbf{V}}_i(t+\Delta t). \tag{S2.7}$$

# S3 Some analytical results for pairwise interactions

## S3.1 Prescribed changes in direction as a function of the relative coordinates of a single group mate in the zonal model

Consider a focal individual $i$ positioned at $(0,0)$ and moving in the positive $x$-direction, parallel to the $x$-axis. When the focal individual $i$ has one partner $j$ in its ZOR then the preferred direction of $i$ is

$$\hat{\mathbf{d}}_{r,i}(t+\Delta t) = \frac{-\hat{\mathbf{r}}_{ij}(t)}{||\hat{\mathbf{r}}_{ij}(t)||}. \tag{S3.1}$$

where $\hat{\mathbf{r}}_{ij} = \frac{\mathbf{x}_j - \mathbf{x}_i}{||\mathbf{x}_j - \mathbf{x}_i||}$.

Let $\mathbf{x}_i = \mathbf{0}$, $\mathbf{x}_j = x\mathbf{i} + y\mathbf{j}$ and $\hat{\mathbf{V}}_i(t) = \mathbf{i}$. Then,

$$\hat{\mathbf{d}}_{r,i}(t+\Delta t) = \frac{-x}{\sqrt{x^2+y^2}}\mathbf{i} - \frac{y}{\sqrt{x^2+y^2}}\mathbf{j}. \tag{S3.2}$$

The magnitude of the change in direction for focal individual $i$ from time $t$ to $t+\Delta t$ can be calculated using the formula

$$\begin{aligned} |\Delta\theta| &= \cos^{-1}\left(\hat{\mathbf{d}}_i(t) \cdot \hat{\mathbf{d}}_{r,i}(t+\Delta t)\right) \\ &= \cos^{-1}\left(\frac{-x}{\sqrt{x^2+y^2}}\right). \end{aligned} \tag{S3.3}$$

In order to determine the sense of rotation of the focal individual as it travels from time $t$ to $t+\Delta t$ we determine

$$\begin{aligned} \operatorname{sgn}\left(\left(\hat{\mathbf{d}}_i(t) \times \hat{\mathbf{d}}_{r,i}(t+\Delta t)\right) \cdot \mathbf{k}\right) &= \operatorname{sgn}\left(\frac{-y}{\sqrt{x^2+y^2}}\right) \\ &= \begin{cases} -1 \text{ if } y > 0, \\ 0 \text{ if } y = 0, \\ 1 \text{ if } y < 0. \end{cases} \end{aligned} \tag{S3.4}$$

A value of $-1$ from equation S3.4 indicates the sense of rotation of the focal individual is clockwise, 0 indicates that $\hat{\mathbf{d}}_i(t)$ and $\hat{\mathbf{d}}_{r,i}(t+\Delta t)$ are parallel and 1 indicates the sense of rotation of the focal individual is anticlockwise. Therefore, the change in direction over time of the focal individual due to a repulsion interaction with a single partner is given by

$$\frac{\Delta\theta}{\Delta t} = \begin{cases} \frac{\mathrm{sgn}(-y)|\Delta\theta|}{\Delta t} & \text{if } \mathrm{sgn}(-y) \neq 0, \\ \frac{|\Delta\theta|}{\Delta t} & \text{if } \mathrm{sgn}(-y) = 0, \end{cases} \tag{S3.5}$$

in radians.

When the focal individual $i$ has one partner $j$ in its ZOA then the preferred direction of $i$ due to attraction to $j$ is

$$\hat{\mathbf{d}}_{a,i}(t+\Delta t) = \frac{\hat{\mathbf{r}}_{ij}(t)}{||\hat{\mathbf{r}}_{ij}(t)||}. \tag{S3.6}$$

where $\hat{\mathbf{r}}_{ij} = \frac{\mathbf{x}_j - \mathbf{x}_i}{||\mathbf{x}_j - \mathbf{x}_i||}$.

Let $\mathbf{x}_i = \mathbf{0}$, $\mathbf{x}_j = x\mathbf{i} + y\mathbf{j}$ and $\hat{\mathbf{d}}_i(t) = \mathbf{i}$. Then

$$\hat{\mathbf{d}}_{a,i}(t+\Delta t) = \frac{x}{\sqrt{x^2+y^2}}\mathbf{i} + \frac{y}{\sqrt{x^2+y^2}}\mathbf{j}. \tag{S3.7}$$

The magnitude of the change in direction for focal individual $i$ from time $t$ to $t+\Delta t$ can be calculated using the formula

$$\begin{aligned} |\Delta\theta| &= \cos^{-1}\left(\hat{\mathbf{d}}_i(t) \cdot \hat{\mathbf{d}}_{a,i}(t+\Delta t)\right) \\ &= \cos^{-1}\left(\frac{x}{\sqrt{x^2+y^2}}\right). \end{aligned} \tag{S3.8}$$

In order to determine the sense of rotation of the focal individual as it travels from time $t$ to $t+\Delta t$ we determine

$$\begin{aligned} \mathrm{sgn}\left(\left(\hat{\mathbf{d}}_i(t) \times \hat{\mathbf{d}}_{a,i}(t+\Delta t)\right) \cdot \mathbf{k}\right) &= \mathrm{sgn}\left(\frac{y}{\sqrt{x^2+y^2}}\right) \\ &= \begin{cases} 1 \text{ if } y > 0, \\ 0 \text{ if } y = 0, \\ -1 \text{ if } y < 0. \end{cases} \end{aligned} \tag{S3.9}$$

Therefore, the change in direction of motion over time of the focal individual due to an attraction interaction with a single partner is given by

$$\frac{\Delta\theta}{\Delta t} = \begin{cases} \frac{\mathrm{sgn}(y)|\Delta\theta|}{\Delta t} & \text{if } \mathrm{sgn}(-y) \neq 0, \\ \frac{|\Delta\theta|}{\Delta t} & \text{if } \mathrm{sgn}(y) = 0, \end{cases} \tag{S3.10}$$

in radians.

Figure SF1 illustrates the change in angle of motion over time of a focal individual, as governed by equations (S3.5) and (S3.10). Parameter values used for the plot were $r_r = 10$,

$r_o = 20$, $r_a = 50$, and $\Delta t = 0.1$. Figure SF1 also illustrates the signs of $\frac{\Delta\theta}{\Delta t}$, emphasising the extents of the regions over which there are repulsion- and attraction-like interactions. If the focal individual's single partner is in the ZOO, then the focal individual will adjust its direction of motion to match that of its partner, with the corresponding turn otherwise independent of the relative $(x, y)$ coordinates of the partner within that region. The turning rates described by equations (S3.5) and (S3.10) are capped at a magnitude of $\theta_{\Delta t}/\Delta t$ (see section S2).

## S3.2 Prescribed changes in speed and direction as a function of the relative coordinates of a single group mate in the ODE model

Consider a focal individual $i$, positioned at $(0, 0)$ and moving in the positive $x$-direction, parallel to the positive $x$-axis. Assume that the focal individual $i$ has one partner $j$. Let $\mathbf{x}_i = x_i\mathbf{i} + y_i\mathbf{j} = \mathbf{0}$, $\mathbf{x}_j = x_j\mathbf{i} + y_j\mathbf{j}$ and $\mathbf{v}_i = u\mathbf{i}$. For the differential equation model [1, 2] used here we have

$$\begin{cases} \frac{d\mathbf{x}_i}{dt} = \mathbf{v}_i, \quad (i = 1, ..., N) \\ \frac{d\mathbf{v}_i}{dt} = (\alpha - \beta|\mathbf{v}_i|^2)\mathbf{v}_i - \frac{1}{N}\sum_{j\neq i} \nabla U(|\mathbf{x}_i - \mathbf{x}_j|), \quad (i = 1, ..., N). \end{cases} \tag{S3.11}$$

since there is only a pair of individuals and $\mathbf{x}_i = \mathbf{0}$ we have $|\mathbf{x}_i - \mathbf{x}_j| = \sqrt{x_j^2 + y_j^2}$ and

$$U\left(\sqrt{x_j^2 + y_j^2}\right) = -C_A e^{\frac{-\sqrt{x_j^2+y_j^2}}{l_A}} + C_R e^{\frac{-\sqrt{x_j^2+y_j^2}}{l_R}}. \tag{S3.12}$$

To compute $\nabla U$, we first calculate:

$$\begin{aligned} \frac{\partial}{\partial x}\left(U\left(\sqrt{x^2+y^2}\right)\right)\Big|_{(x,y)=(x_j,y_j)} &= \frac{C_A x_j}{l_A}\left(x_j^2+y_j^2\right)^{-\frac{1}{2}} e^{-\frac{\sqrt{\left(x_j^2+y_j^2\right)}}{l_A}} - \frac{C_R x_j}{l_R}\left(x_j^2+y_j^2\right)^{-\frac{1}{2}} e^{-\frac{\sqrt{\left(x_j^2+y_j^2\right)}}{l_R}}, \\ \frac{\partial}{\partial y}\left(U\left(\sqrt{x^2+y^2}\right)\right)\Big|_{(x,y)=(x_j,y_j)} &= \frac{C_A y_j}{l_A}\left(x_j^2+y_j^2\right)^{-\frac{1}{2}} e^{-\frac{\sqrt{\left(x_j^2+y_j^2\right)}}{l_A}} - \frac{C_R y_j}{l_R}\left(x_j^2+y_j^2\right)^{-\frac{1}{2}} e^{-\frac{\sqrt{\left(x_j^2+y_j^2\right)}}{l_R}}. \end{aligned} \tag{S3.13}$$

In component form, the equation describing changes in velocity (S3.11) can be written as:

$$\begin{aligned} \frac{du_i(t)}{dt} &= \left(\alpha - \beta u(t)^2\right)u(t) - \frac{1}{2}\frac{\partial}{\partial x}\left(U\left(\sqrt{x^2+y^2}\right)\right)\Big|_{(x,y)=(x_j,y_j)}, \\ \frac{dv_i(t)}{dt} &= -\frac{1}{2}\frac{\partial}{\partial y}\left(U\left(\sqrt{x^2+y^2}\right)\right)\Big|_{(x,y)=(x_j,y_j)}. \end{aligned} \tag{S3.14}$$

Now since the focal individual is positioned at the center and moving in the direction of the positive $x-$axis, we define the speed of the focal individual to be some function of $t$

say $s(t)$ and the orientation of the focal individual to be $\theta(t) = 0$. The components of the velocity of the focal individual can be written as

$$\begin{aligned} u_i(t) &= s(t)\cos(\theta(t)), \\ v_i(t) &= s(t)\sin(\theta(t)). \end{aligned} \tag{S3.15}$$

Differentiating equtions (S3.15) with respect to $t$ gives

$$\begin{aligned} \frac{du_i(t)}{dt} &= \cos(\theta(t))\frac{ds(t)}{dt} - s(t)\sin(\theta(t))\frac{d\theta(t)}{dt}, \\ \frac{dv_i(t)}{dt} &= \sin(\theta(t))\frac{ds(t)}{dt} + s(t)\cos(\theta(t))\frac{d\theta(t)}{dt}. \end{aligned} \tag{S3.16}$$

Then, since $\theta(t) = 0$,

$$\begin{aligned} \frac{du_i(t)}{dt} &= \frac{ds(t)}{dt}, \\ \frac{dv_i(t)}{dt} &= s(t)\frac{d\theta(t)}{dt}. \end{aligned} \tag{S3.17}$$

Comparing equations (S3.14) and (S3.17) and substituting (S3.13) gives

$$\frac{ds(t)}{dt} = \left(\alpha - \beta\left(u(t)^2\right)\right)u(t) - \frac{1}{2}\left(\frac{C_A x_j}{l_A}\left(x_j^2 + y_j^2\right)^{-\frac{1}{2}} e^{-\frac{\sqrt{\left(x_j^2+y_j^2\right)}}{l_A}} - \frac{C_R x_j}{l_R}\left(x_j^2 + y_j^2\right)^{-\frac{1}{2}} e^{-\frac{\sqrt{\left(x_j^2+y_j^2\right)}}{l_R}}\right) \tag{S3.18}$$

and

$$\frac{d\theta(t)}{dt} = -\frac{1}{2s(t)}\left(\frac{C_A y_j}{l_A}\left(x_j^2 + y_j^2\right)^{-\frac{1}{2}} e^{-\frac{\sqrt{\left(x_j^2+y_j^2\right)}}{l_A}} - \frac{C_R y_j}{l_R}\left(x_j^2 + y_j^2\right)^{-\frac{1}{2}} e^{-\frac{\sqrt{\left(x_j^2+y_j^2\right)}}{l_R}}\right). \tag{S3.19}$$

Equations (S3.18) and (S3.19) describe the change in speed over time and change in angle of motion over time of the focal individual relative to one partner respectively.

Figures SF2 and SF3 illustrate $\frac{ds}{dt}$ and $\frac{d\theta}{dt}$, as defined by equations (S3.18) and (S3.19), with $\alpha = 0.15$, $\beta = 0.05$, $C_A = 100$, $C_R = 50$, $l_A = 100$, $l_R = 20$, $N = 2$, $u = 0.75$, $v = 0$, and hence $s = 0.75$. Figures SF4 and SF5 illustrate the signs of $\frac{ds}{dt}$, and $\frac{d\theta}{dt}$, emphasising the extents of the regions over which there are repulsion- and attraction-like interactions.

The pairwise interactions for this model resemble the repulsion and attraction behaviour observed among shoaling fish. In particular, three species of fish (golden shiners (*Notemigonus crysoleucas*), eastern mosquitofish (*Gambusia holbrooki*), and x-ray tetras (*Pristella maxillaris*) have been observed to adjust their speed as a function of the relative locations of their partners following a very similar pattern to that in Figure SF2 [3, 4, 5]. Both golden shiners and x-ray tetras exhibit similar turning behaviour as a function of the relative coordinates of their groupmates to that illustrated in Figure SF3, with tendencies to turn away from very near neighbours and towards those that are further away [3, 5]. Eastern mosquitofish have been observed to turn towards far neighbours, but employ more complex patterns of turning in response to near neighbours than that in Figure SF3 [4, 6].

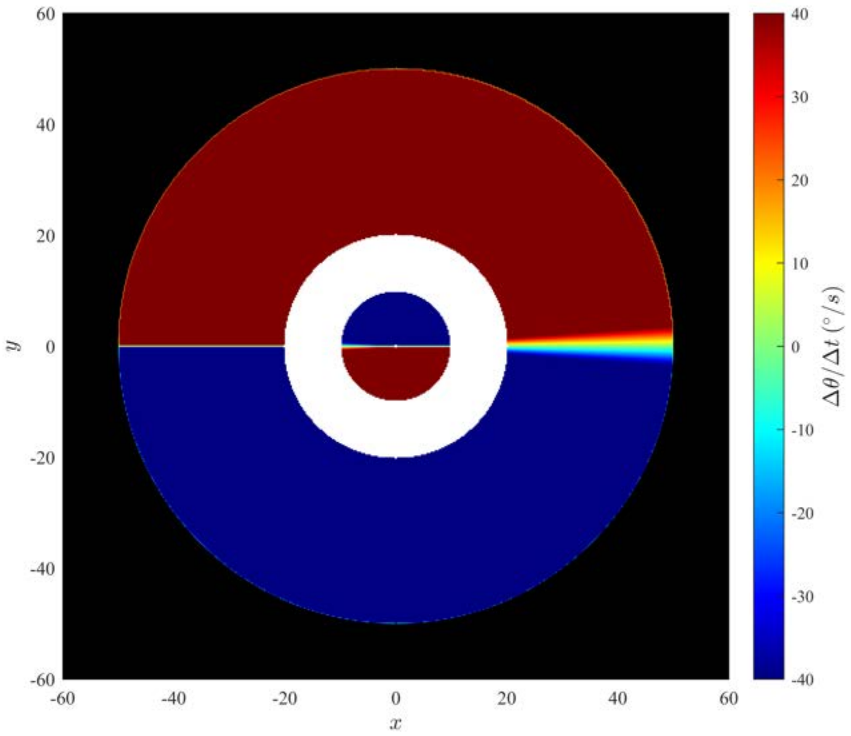


**Fig SF1.** Constrained changes in angle of motion as a function of the relative coordinates of a single partner, as defined by equations (S3.5) and (S3.10), but with a maximum turning rate of 40° per second imposed. A focal individual is located at the origin moving right parallel to the $x$-axis. Positive changes in angle of motion indicate a turn to the left by the focal individual (relative to its direction of motion), whereas negative changes in angle of motion indicate a turn to the right. When the partner is far to the left of the focal individual, then the focal individual turns left (positive region) and when the partner is far to the right of the focal individual, then the focal individual turns right (negative region). This reflects the model based rule of attraction, moderated by changes in direction. If partner is close and to the left of the focal individual then the focal individual turns away by turning right and if the partner is close and to the right of the focal individual then the focal individual turns away by turning left. This behaviour reflects the model prescribed rules for repulsion by individuals at close range. In the orientation zone (white region) the focal individual will act to match the direction of motion of its partner, irrespective of the relative coordinates of its partner within that zone. Outside the attraction zone (black region) the focal individual has no interaction with its partner.

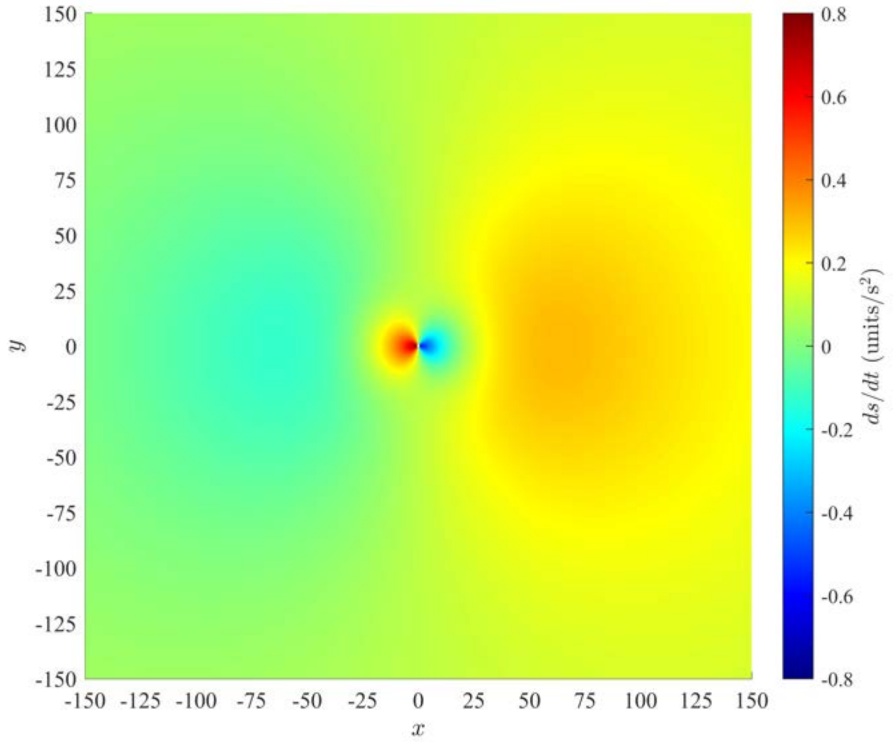


**Fig SF2.** Changes in speed as a function of the relative coordinates of a single partner, as defined by equation (S3.18) in the ODE model. A focal individual is located at the origin moving right parallel to the $x$-axis. When the focal individual has a partner in front at close range then the focal individual slows down (negative change in speed over time) and when the focal individual has a partner behind at close range then the focal individual speeds up (positive change in speed over time). Thus the differential equation model prescribes speed mediated collision avoidance at short range. When the focal individual has a partner in front at greater distances then the focal individual speeds up (positive change in speed over time) and when the focal individual has a partner behind at greater distances then the focal individual slows down (negative change in speed over time). Thus the differential equation model prescribes speed mediated attraction-like behaviour in the far field.

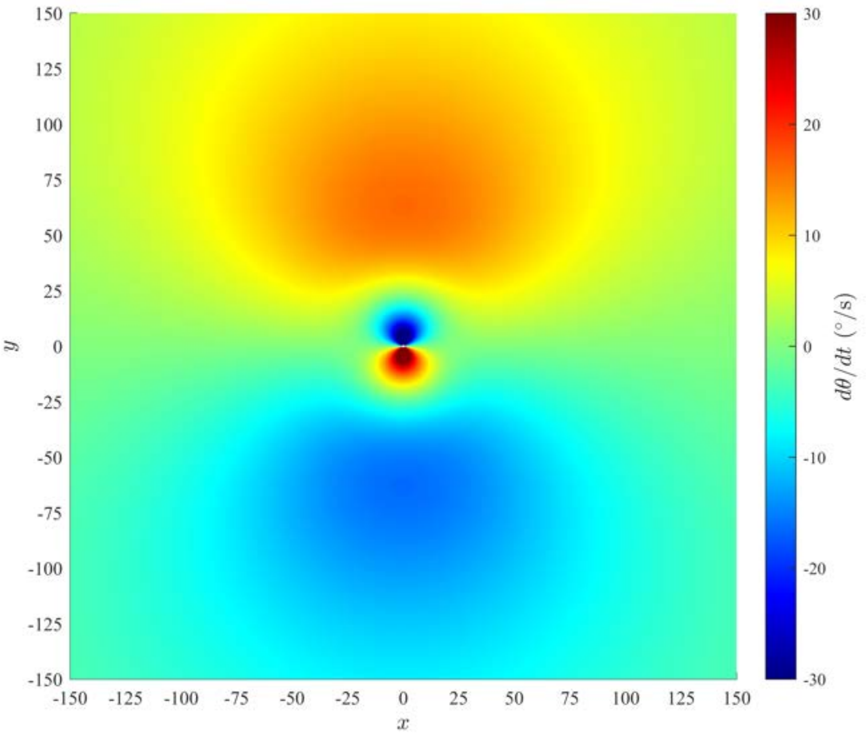


**Fig SF3.** Changes in angle of motion as a function of the relative coordinates of a single partner, as defined by equation (S3.19) in the ODE model. A focal individual is located at the origin moving right parallel to the $x$-axis. Positive changes in angle of motion indicate a turn to the left by the focal individual (relative to its direction of motion), whereas negative changes in angle of motion indicate a turn to the right.

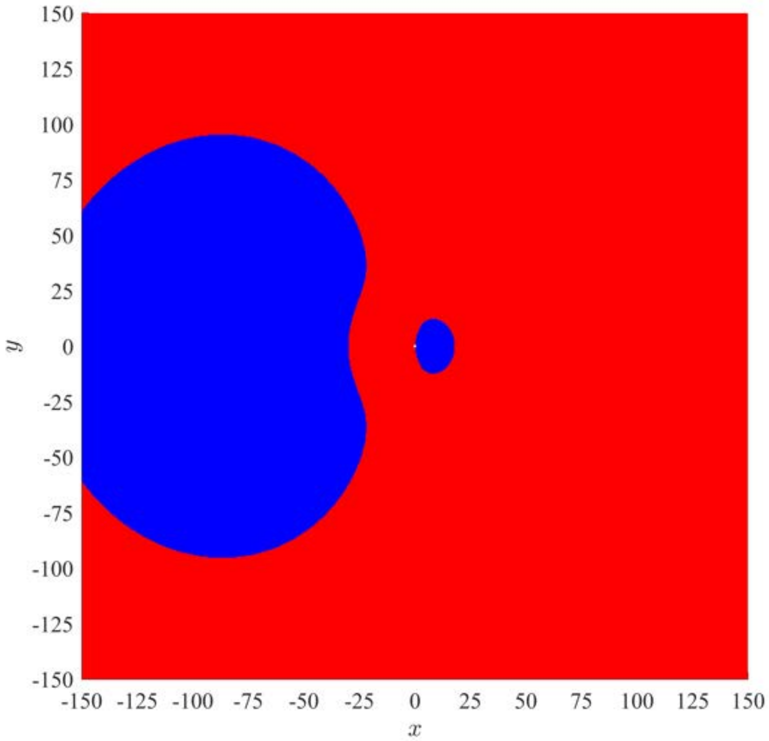


**Fig SF4.** The sign of changes in speed as a function of the relative coordinates of a single partner, as defined by the ODE model. A focal individual is located at the origin moving right parallel to the $x$-axis. When the single partner occupies a blue region in the graph, then the focal individual slows down. If the partner occupies a red region, then the focal individual increases its speed.

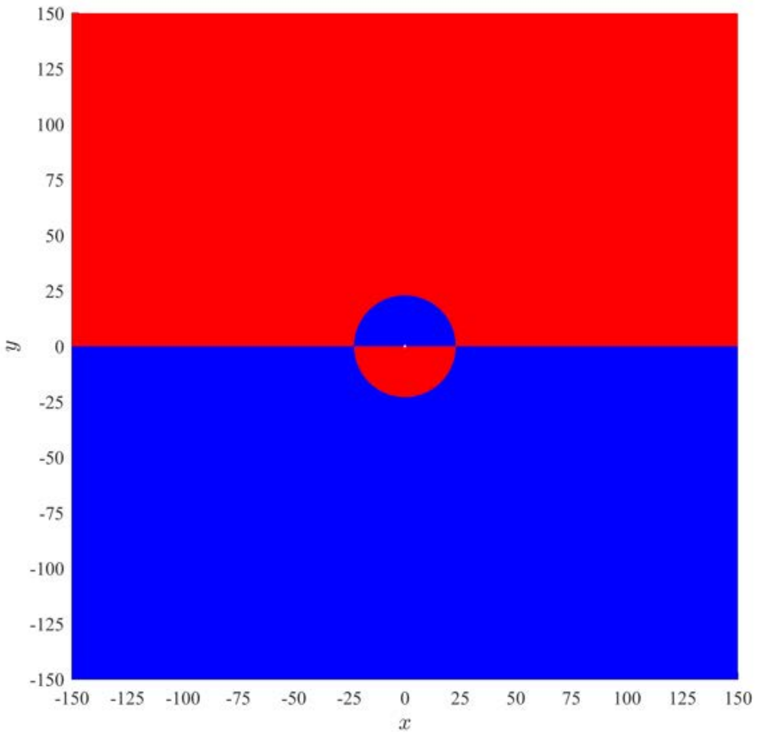


**Fig SF5.** Sense of rotation of turns as a function of the relative coordinates of a single partner, as defined by the ODE model. A focal individual is located at the origin moving right parallel to the $x$-axis. Positive changes in angle of motion indicate a turn to the left (red region) by the focal individual (relative to its direction of motion), whereas negative changes in angle of motion indicate a turn to the right (blue region).

# S4 Analysis of group fragmentation and algorithmic categorisation of emergent states

We classified the emergent states of short (1000 time step) duration simulations of the zonal model and simulations of the ODE model via visual inspection. However, for longer duration simulations of the zonal model, we applied algorithms to identify the fragmentation of groups, and to classify the emergent behaviour of non-fragmenting groups.

We identified group fragmentation with the help of the algorithms described in [7, 8] for identifying the formation of subgroups. In short, individuals were identified as being a member of a particular subgroup if they were within a threshold distance of any other member of that subgroup. We set the threshold for subgroup membership at 14 units, equal to the outer radius of the zone of attraction in all our calculations. A full group was treated as not having fragmented if the largest subgroup retained 95% or more of the simulated individuals throughout the second half of a simulation.

If a group did not fragment, then we classified its emergent behaviour via summaries of the group's polarisation and angular momentum over the second half of the corresponding simulation. The group polarisation at a particular time $t$ was determined via:

$$p(t) = \frac{1}{N}\left|\left|\sum_{i=1}^{N} \hat{\mathbf{V}}_i(t)\right|\right|, \tag{S4.1}$$

where $\hat{\mathbf{V}}_i(t)$ was the unit vector pointing in the direction of motion of individual $i$ (at time $t$). Polarisation measures the agreement in the directions of motion of individuals, with values of $p(t)$ close to 1 indicating near perfect alignment in movement, and values of $p(t)$ closer to zero coinciding with a greater scatter in directions of motion. We then took the average of $p(t)$ over the second half of a simulation to give $p_{group}$.

The angular momentum order parameter is a measure of the degree of agreement between individuals in terms of rotating in a common sense/direction about the group centre. For our calculations, we identified the group centre, written in vector form as $\mathbf{c}_{group}(t)$, by averaging the $x$- and $y$-coordinates of all individuals at time $t$. Then, the angular momentum at time $t$ was determined by:

$$m(t) = \frac{1}{N}\left|\left|\sum_{i=1}^{N} \frac{\mathbf{r}_{ci} \times \mathbf{V}_i(t)}{||\mathbf{r}_{ci} \times \mathbf{V}_i(t)||}\right|\right|, \tag{S4.2}$$

where $\mathbf{r}_{ci} = \mathbf{c}_i(t) - \mathbf{c}_{group}(t)$ is the vector pointing from the group centre to the position of individual $i$ (at $\mathbf{c}_i(t) = x_i(t)\mathbf{i} + y_i(t)\mathbf{j}$). We then took the average of $m(t)$ over the second half of a simulation to give $m_{group}$.

We then applied the scheme used in [9] to classify emergent patterns of motion. As noted in the main text, groups that did not fragment with $p_{group} > 0.65$ and $m_{group} < 0.35$ were classified as exhibiting parallel aligned movement, and swarming ($p_{group} < 0.35$, $m_{group} < 0.35$) and milling ($p_{group} < 0.35$, $m_{group} > 0.65$) groups were classified together as exhibiting cohesion without parallel movement.

# S5 Supplementary plots for analysis of zonal model simulations with 1000 time steps

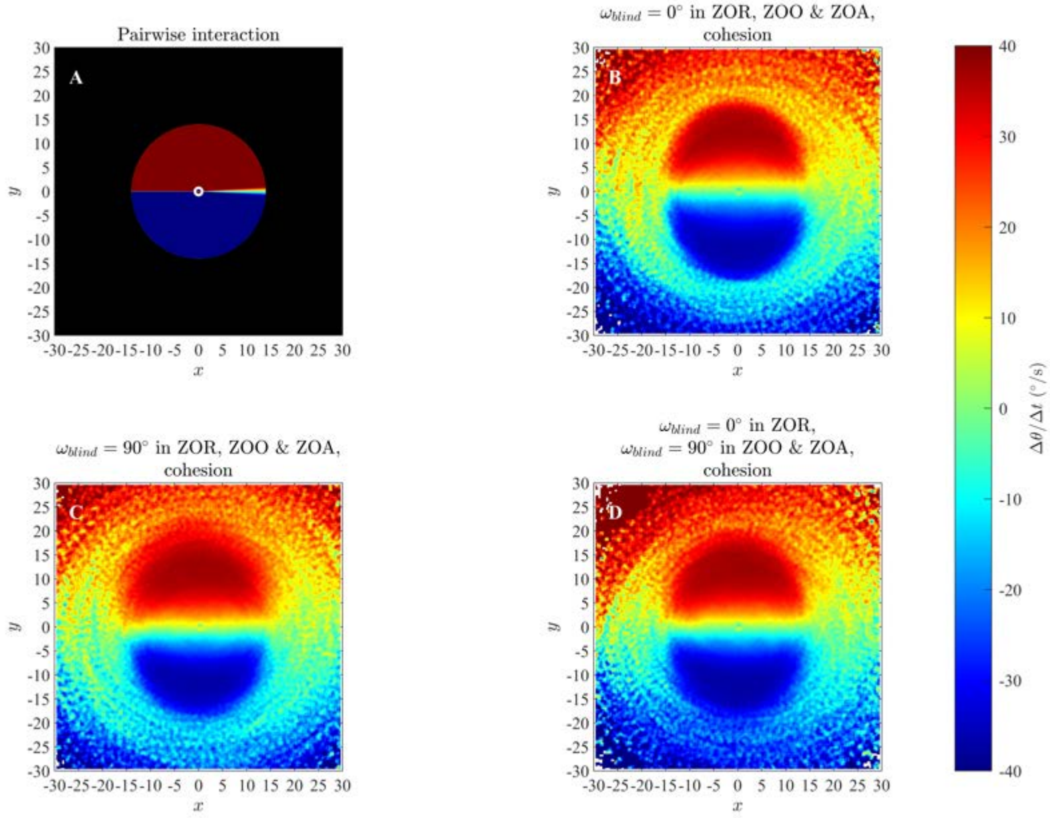


**Fig SF6.** Panel A: analytical pairwise interactions for given parameter values, as described in section S3.1, where turning of the individuals is governed by equations (S3.5) and (S3.10). Panels B, C and D illustrate changes in direction of motion of individuals as a function of the relative positions of partners obtained via analysis of simulations with $r_r = 0.5$, $\Delta r_o = 0.51$ and $\Delta r_a = 12.99$ using the averaging method. The focal individual is located at the origin moving right parallel to the $x$-axis. (Derived from simulations with $N = 25$ individuals.)

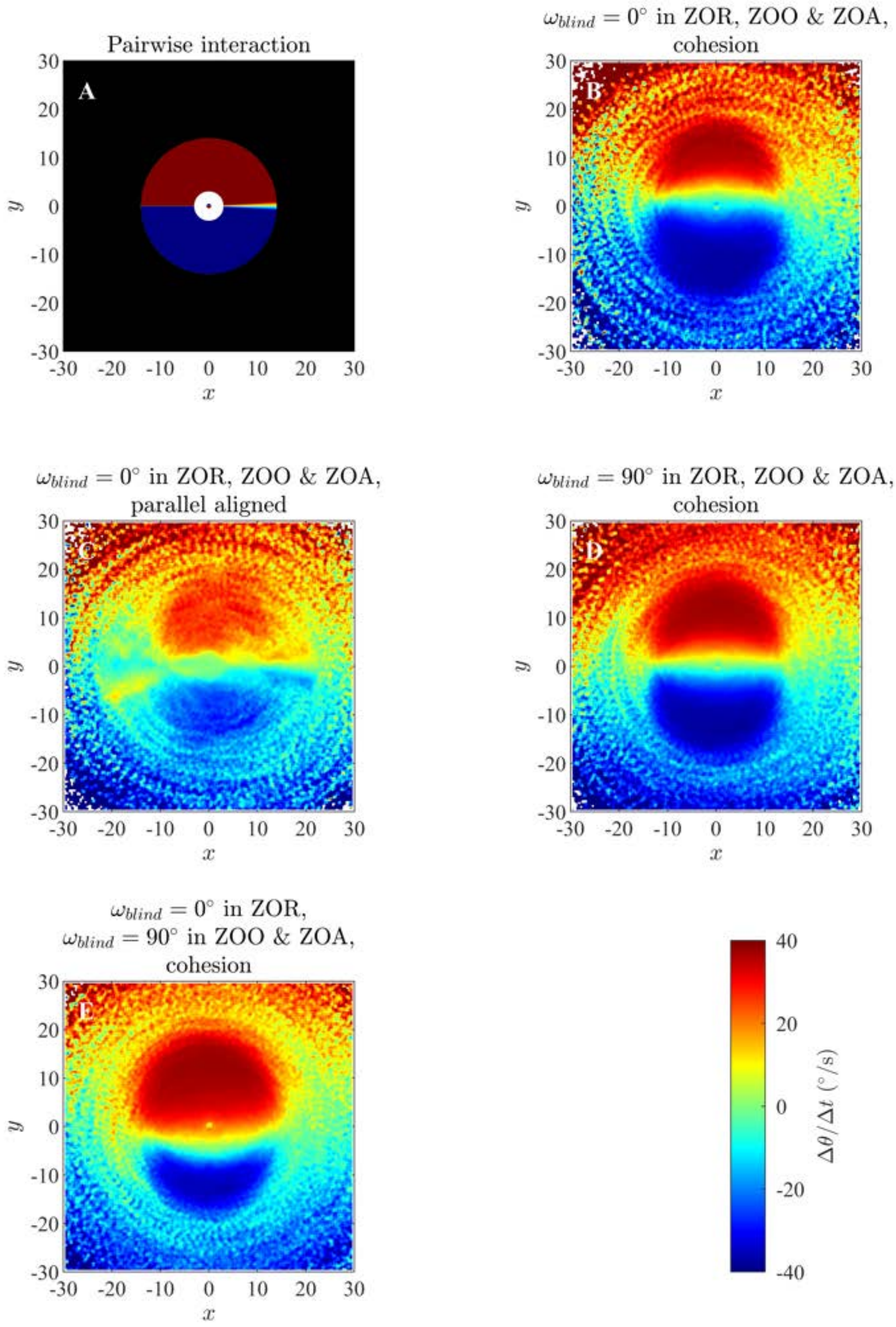


**Fig SF7.** Panel A: analytical pairwise interactions for given parameter values, as described in section S3.1, where turning of the individuals is governed by equations (S3.5) and (S3.10). Panels B, C, D and E illustrate changes in direction of motion of individuals as a function of the relative positions of partners obtained via analysis of simulations with $r_r = 0.5$, $\Delta r_o = 2.5$ and $\Delta r_a = 11$ using the averaging method. (Derived from simulations with $N = 25$ individuals.)

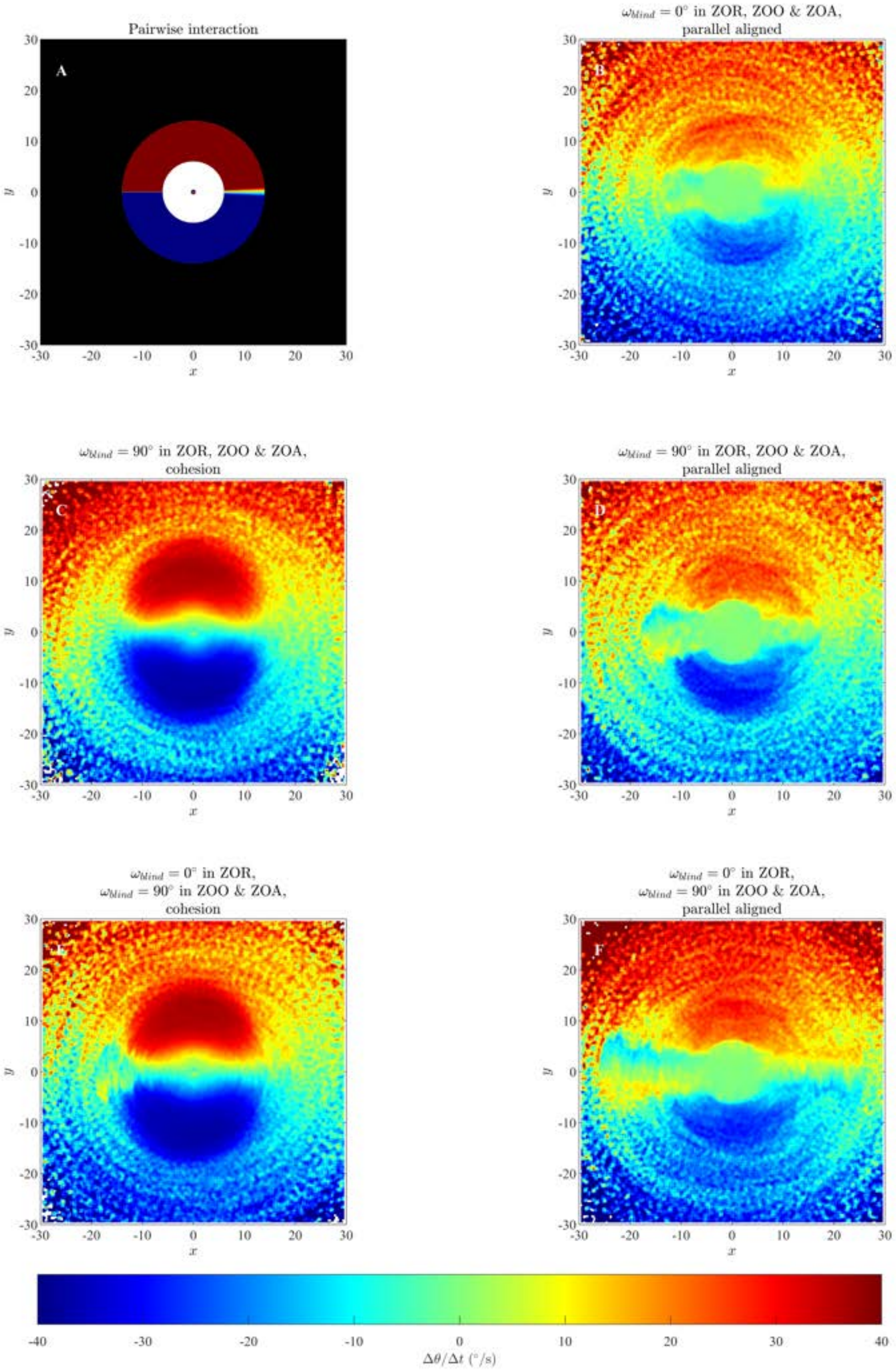


**Fig SF8.** Panel A: analytical pairwise interactions for given parameter values, as described in section S3.1, where turning of the individuals is governed by equations (S3.5) and (S3.10). Panels B, C, D, E and F illustrate changes in direction of motion of individuals as a function of the relative positions of partners obtained via analysis of simulations with $r_r = 0.5$, $\Delta r_o = 5.5$ and $\Delta r_a = 8$ using the averaging method. (Derived from simulations with $N = 25$ individuals.)

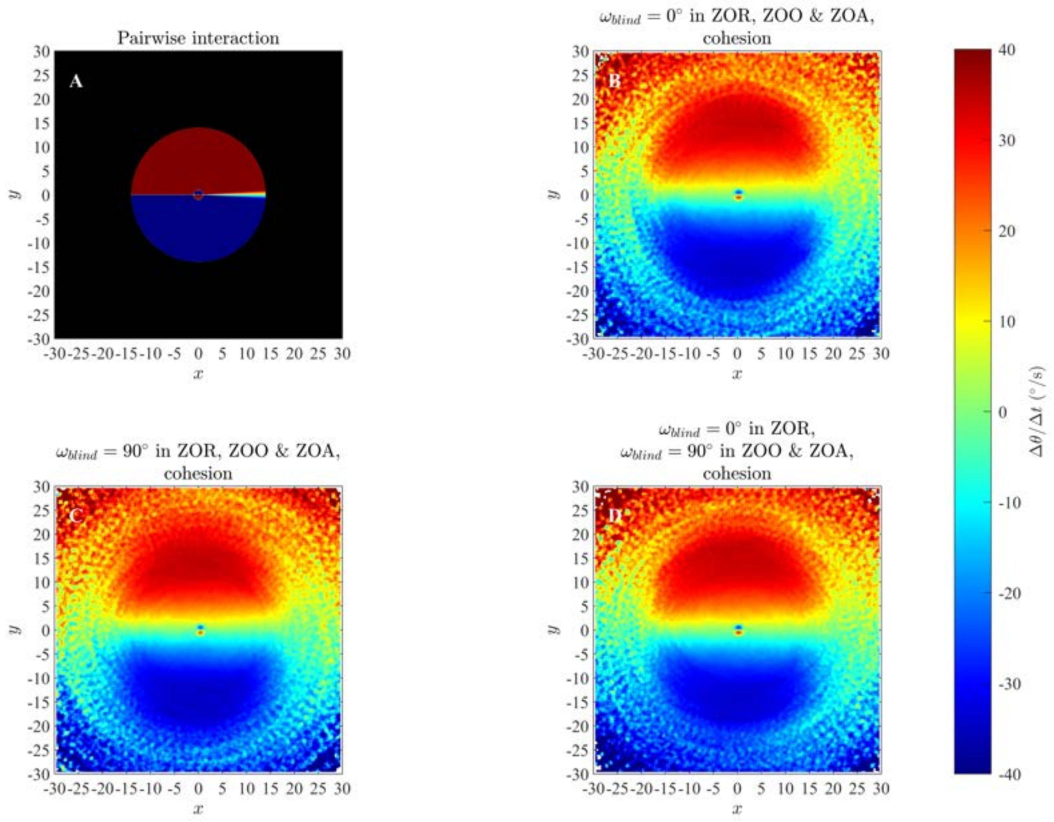


**Fig SF9.** Panel A: analytical pairwise interactions for given parameter values, as described in section S3.1, where turning of the individuals is governed by equations (S3.5) and (S3.10). Panels B, C and D illustrate changes in direction of motion of individuals as a function of the relative positions of partners obtained via analysis of simulations with $r_r = 1$, $\Delta r_o = 0.01$ and $\Delta r_a = 12.99$ using the averaging method. (Derived from simulations with $N = 25$ individuals.)

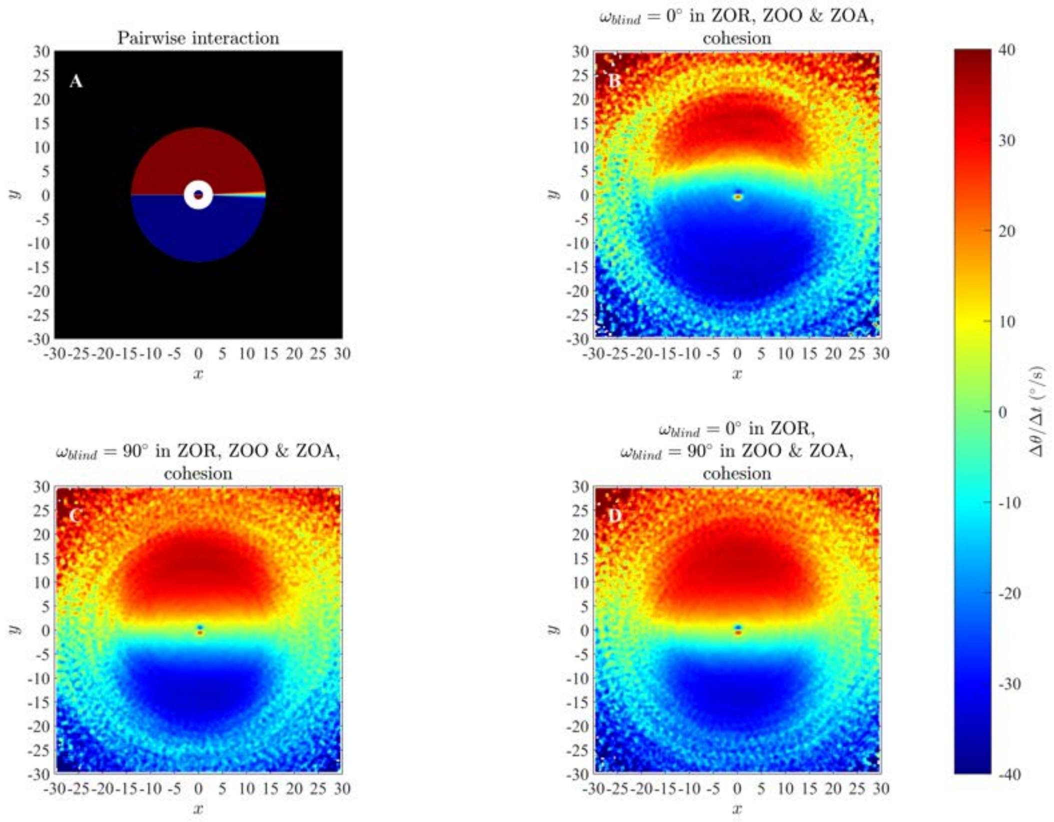


**Fig SF10.** Panel A: analytical pairwise interactions for given parameter values, as described in section S3.1, where turning of the individuals is governed by equations (S3.5) and (S3.10). Panels B, C and D illustrate changes in direction of motion of individuals as a function of the relative positions of partners obtained via analysis of simulations with $r_r = 1$, $\Delta r_o = 2$ and $\Delta r_a = 11$ using the averaging method. (Derived from simulations with $N = 25$ individuals.)

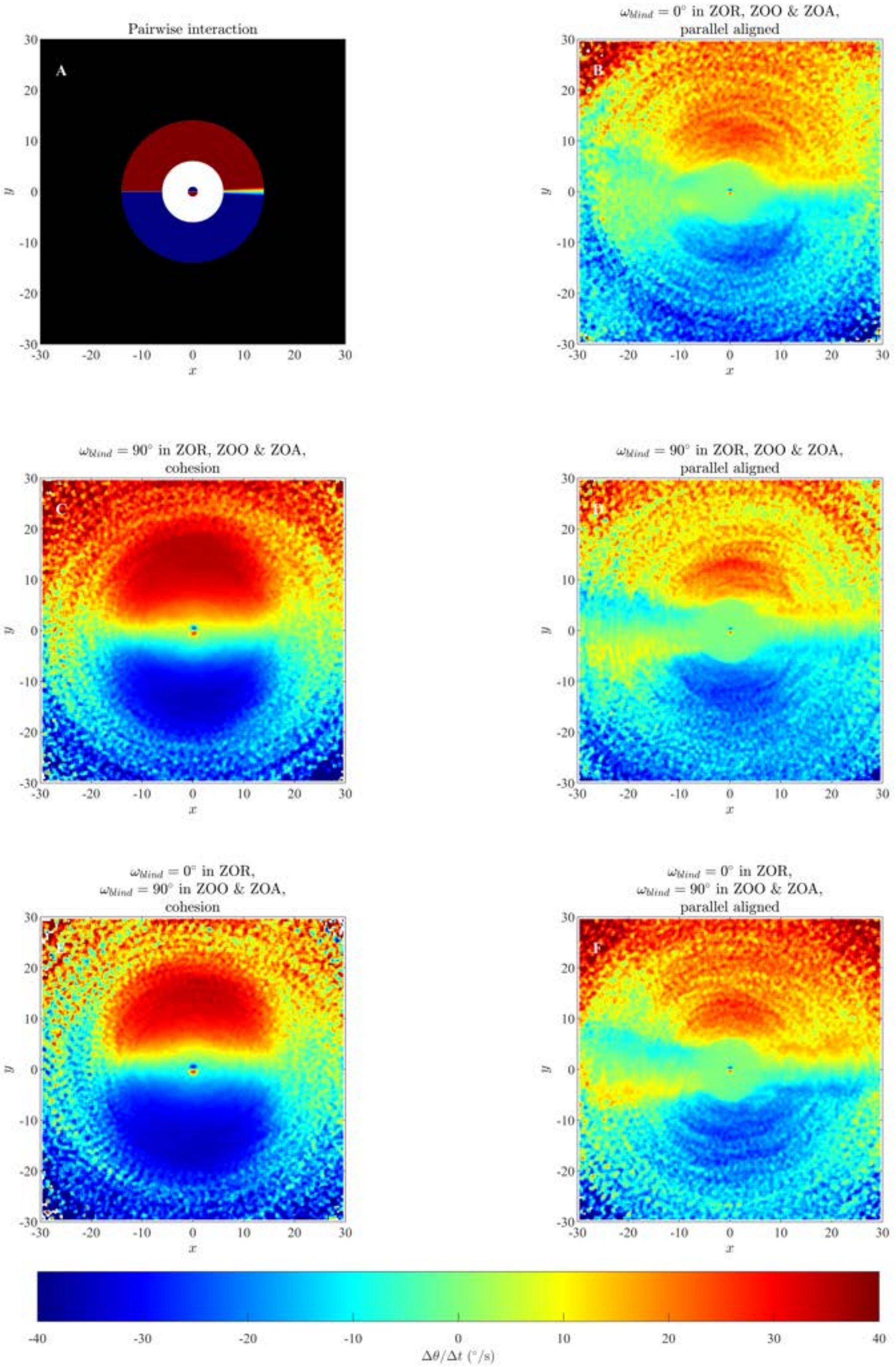


**Fig SF11.** Panel A: analytical pairwise interactions for given parameter values, as described in section S3.1, where turning of the individuals is governed by equations (S3.5) and (S3.10). Panels B, C, D, E and F illustrate changes in direction of motion of individuals as a function of the relative positions of partners obtained via analysis of simulations with $r_r = 1$, $\Delta r_o = 5$ and $\Delta r_a = 8$ using the averaging method. (Derived from simulations with $N = 25$ individuals.)

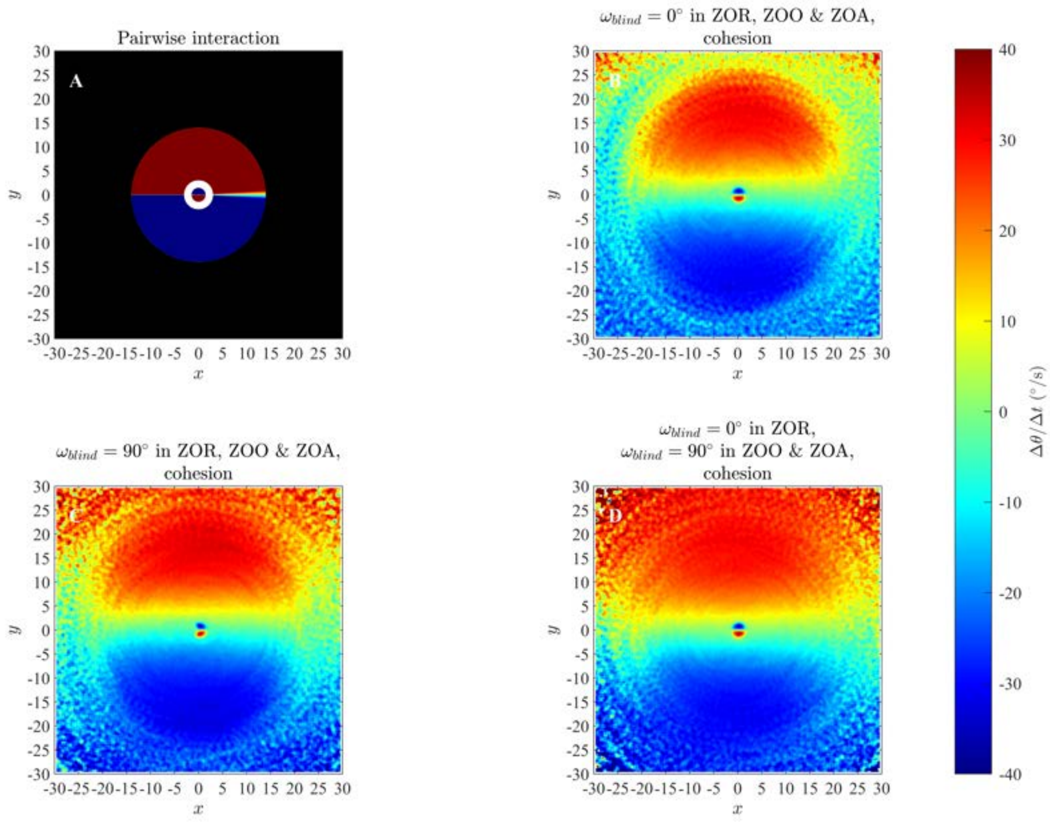


**Fig SF12.** Panel A: analytical pairwise interactions for given parameter values, as described in section S3.1, where turning of the individuals is governed by equations (S3.5) and (S3.10). Panels B, C and D illustrate changes in direction of motion of individuals as a function of the relative positions of partners obtained via analysis of simulations with $r_r = 1.5$, $\Delta r_o = 1.5$ and $\Delta r_a = 11$ using the averaging method. (Derived from simulations with $N = 25$ individuals.)

# S6 Simulations and plots for zonal model for 10000 time steps

Table ST1 lists the data subsets and their emergent collective behaviour for simulations run over 10000 time steps. Corresponding results of analysis appear in Figures SF13 to SF21.

**Table ST1.** Summary of zone size, size of blind region and the respective emergent collective behaviour for simulations run over 10000 time steps.

| $r_r$ | $\Delta r_o$ | $\Delta r_a$ | Form of blind zone as given | Emergent Pattern |
|---|---|---|---|---|
| 0.5 | 0.51 | 12.99 | In ZOO, ZOA and ZOR $\omega_{blind} = 90°$ | cohesion/ fragmented |
| 0.5 | 2.5 | 11 | In ZOO, ZOA and ZOR $\omega_{blind} = 90°$ | cohesion/ parallel aligned/ fragmented |
| 0.5 | 5.5 | 8 | In ZOO, ZOA and ZOR $\omega_{blind} = 90°$ | cohesion/ parallel aligned/ fragmented |
| 1 | 0.01 | 12.99 | In ZOO, ZOA and ZOR $\omega_{blind} = 90°$ | cohesion/ fragmented |
| 1 | 2 | 11 | In ZOO, ZOA and ZOR $\omega_{blind} = 90°$ | cohesion/ fragmented |
| 1 | 5 | 8 | In ZOO, ZOA and ZOR $\omega_{blind} = 90°$ | cohesion/ parallel aligned/ fragmented |
| 1.5 | 1.5 | 11 | In ZOO, ZOA and ZOR $\omega_{blind} = 90°$ | cohesion/ fragmented |
| 1.5 | 4.5 | 8 | In ZOO, ZOA and ZOR $\omega_{blind} = 90°$ | cohesion/ parallel aligned/ fragmented |
| 2 | 1 | 11 | In ZOO, ZOA and ZOR $\omega_{blind} = 90°$ | fragmented |
| 0.5 | 0.51 | 12.99 | In ZOO, ZOA and ZOR $\omega_{blind} = 0°$ | cohesion/ fragmented |
| 0.5 | 2.5 | 11 | In ZOO, ZOA and ZOR $\omega_{blind} = 0°$ | cohesion/ parallel aligned/ fragmented |
| 0.5 | 5.5 | 8 | In ZOO, ZOA and ZOR $\omega_{blind} = 0°$ | parallel aligned/ fragmented |
| 1 | 0.01 | 12.99 | In ZOO, ZOA and ZOR $\omega_{blind} = 0°$ | cohesion/ fragmented |
| 1 | 2 | 11 | In ZOO, ZOA and ZOR $\omega_{blind} = 0°$ | cohesion/ fragmented |
| 1 | 5 | 8 | In ZOO, ZOA and ZOR $\omega_{blind} = 0°$ | parallel aligned/ fragmented |
| 1.5 | 1.5 | 11 | In ZOO, ZOA and ZOR $\omega_{blind} = 0°$ | cohesion/ fragmented |
| 1.5 | 4.5 | 8 | In ZOO, ZOA and ZOR $\omega_{blind} = 0°$ | cohesion/ parallel aligned/ fragmented |
| 2 | 1 | 11 | In ZOO, ZOA and ZOR $\omega_{blind} = 0°$ | cohesion/ fragmented |
| 0.5 | 0.51 | 12.99 | In ZOO, ZOA $\omega_{blind} = 90°$, in ZOR $\omega_{blind} = 0°$ | cohesion/ fragmented |
| 0.5 | 2.5 | 11 | In ZOO, ZOA $\omega_{blind} = 90°$, in ZOR $\omega_{blind} = 0°$ | cohesion/ parallel aligned/ fragmented |
| 0.5 | 5.5 | 8 | In ZOO, ZOA $\omega_{blind} = 90°$, in ZOR $\omega_{blind} = 0°$ | cohesion/ parallel aligned/ fragmented |
| 1 | 0.01 | 12.99 | In ZOO, ZOA $\omega_{blind} = 90°$, in ZOR $\omega_{blind} = 0°$ | cohesion/ fragmented |
| 1 | 2 | 11 | In ZOO, ZOA $\omega_{blind} = 90°$, in ZOR $\omega_{blind} = 0°$ | cohesion/ fragmented |
| 1 | 5 | 8 | In ZOO, ZOA $\omega_{blind} = 90°$, in ZOR $\omega_{blind} = 0°$ | cohesion/ parallel aligned/ fragmented |
| 1.5 | 1.5 | 11 | In ZOO, ZOA $\omega_{blind} = 90°$, in ZOR $\omega_{blind} = 0°$ | cohesion/ fragmented |
| 1.5 | 4.5 | 8 | In ZOO, ZOA $\omega_{blind} = 90°$, in ZOR $\omega_{blind} = 0°$ | cohesion/ parallel aligned/ fragmented |
| 2 | 1 | 11 | In ZOO, ZOA $\omega_{blind} = 90°$, in ZOR $\omega_{blind} = 0°$ | fragmented |

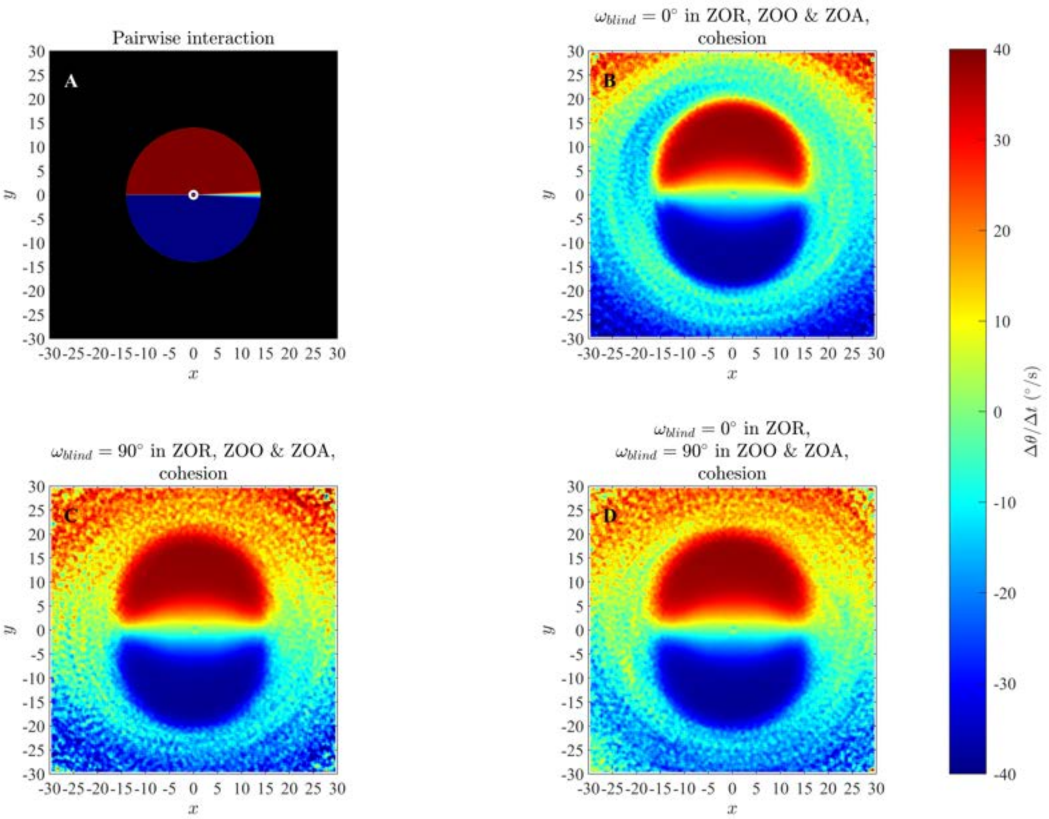


**Fig SF13.** Panel A: analytical pairwise interactions for given parameter values, as described in section S3.1, where turning of the individuals is governed by equations (S3.5) and (S3.10). Panels B, C and D illustrate changes in direction of motion of individuals as a function of the relative positions of partners obtained via analysis of simulations with $r_r = 0.5$, $\Delta r_o = 0.51$ and $\Delta r_a = 12.99$ using the averaging method. The focal individual is located at the origin moving right parallel to the $x$-axis. Positive changes in angle of motion indicate a turn to the left by the focal individual, whereas negative changes in angle of motion indicate a turn to the right. Here $N = 25$ and simulations were run for 10000 time steps.

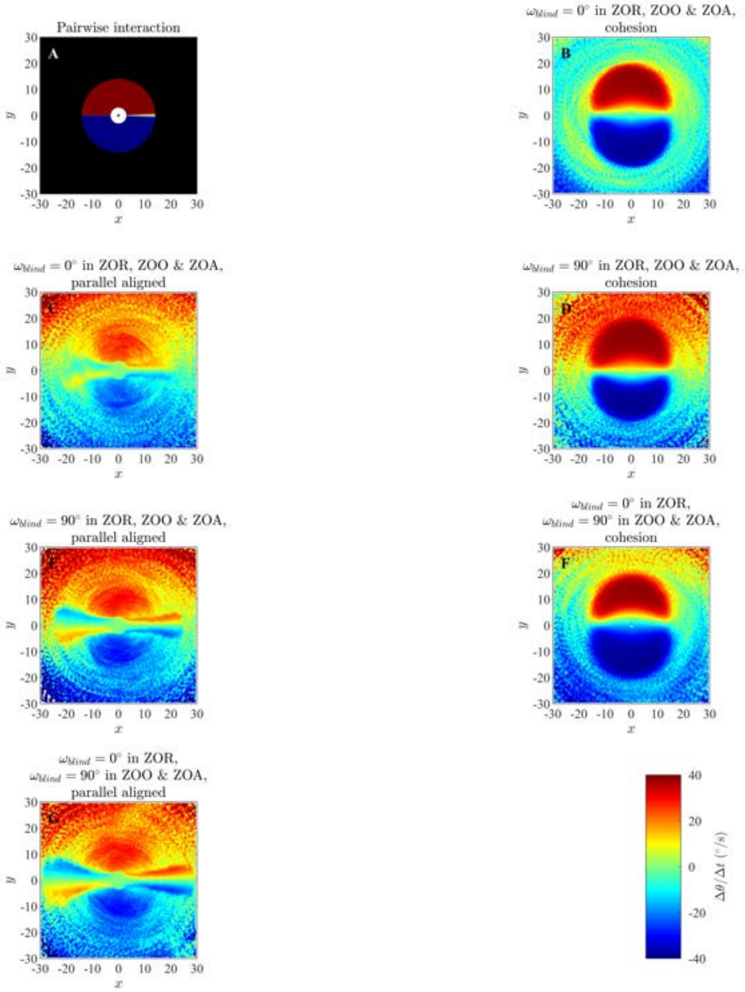


**Fig SF14.** Panel A: analytical pairwise interactions for given parameter values, as described in section S3.1, where turning of the individuals is governed by equations (S3.5) and (S3.10). Panels B, C, D, E, F and G illustrate changes in direction of motion of individuals as a function of the relative positions of partners obtained via analysis of simulations with $r_r = 0.5$, $\Delta r_o = 2.5$ and $\Delta r_a = 11$ using the averaging method. ($N = 25$, 10000 time steps per simulation.)

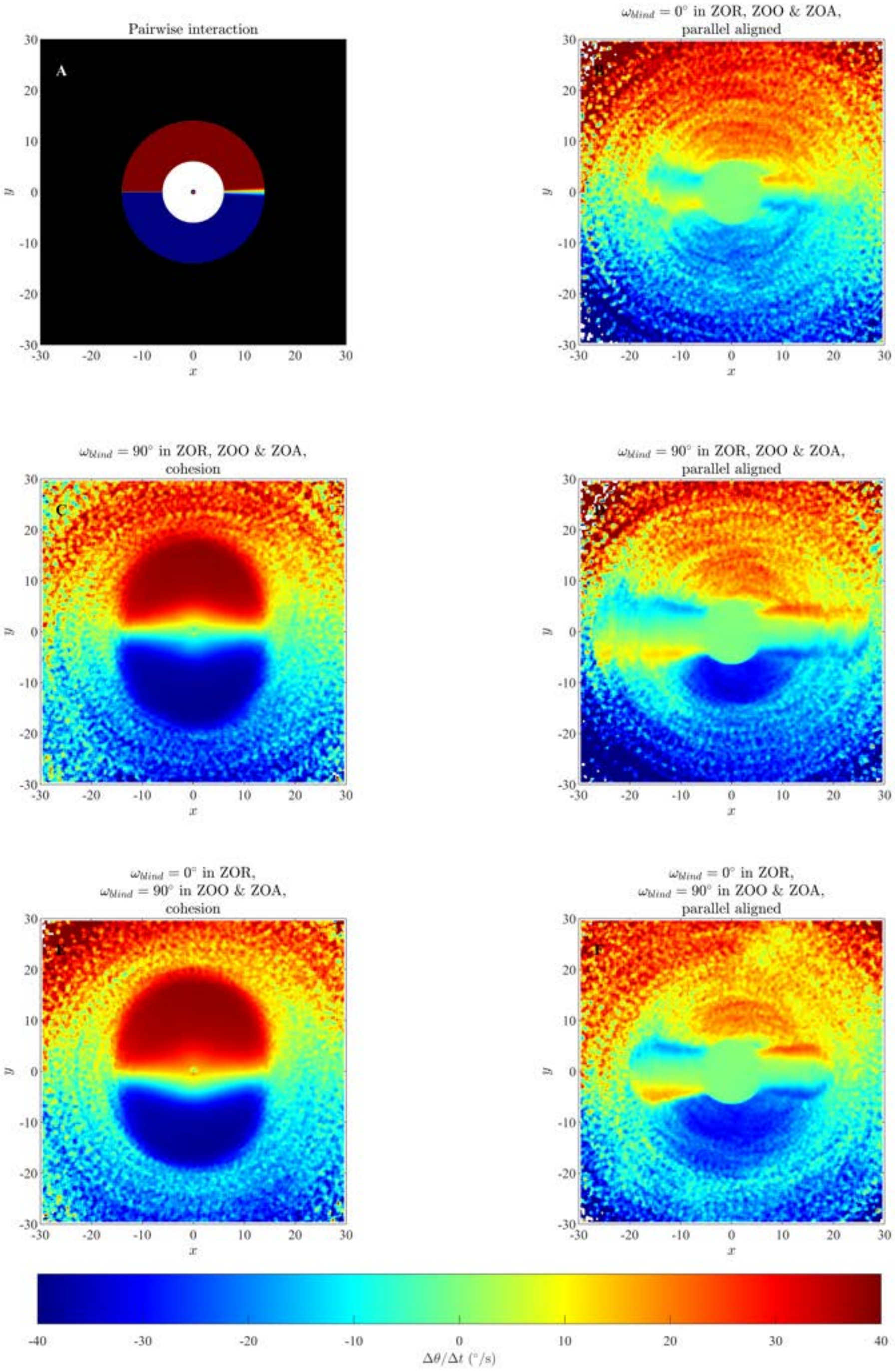


**Fig SF15.** Panel A: analytical pairwise interactions for given parameter values, as described in section S3.1, where turning of the individuals is governed by equations (S3.5) and (S3.10). Panels B, C, D, E and F illustrate changes in direction of motion of individuals as a function of the relative positions of partners obtained via analysis of simulations with $r_r = 0.5$, $\Delta r_o = 5.5$ and $\Delta r_a = 8$ using the averaging method. ($N = 25$, 10000 time steps per simulation.)

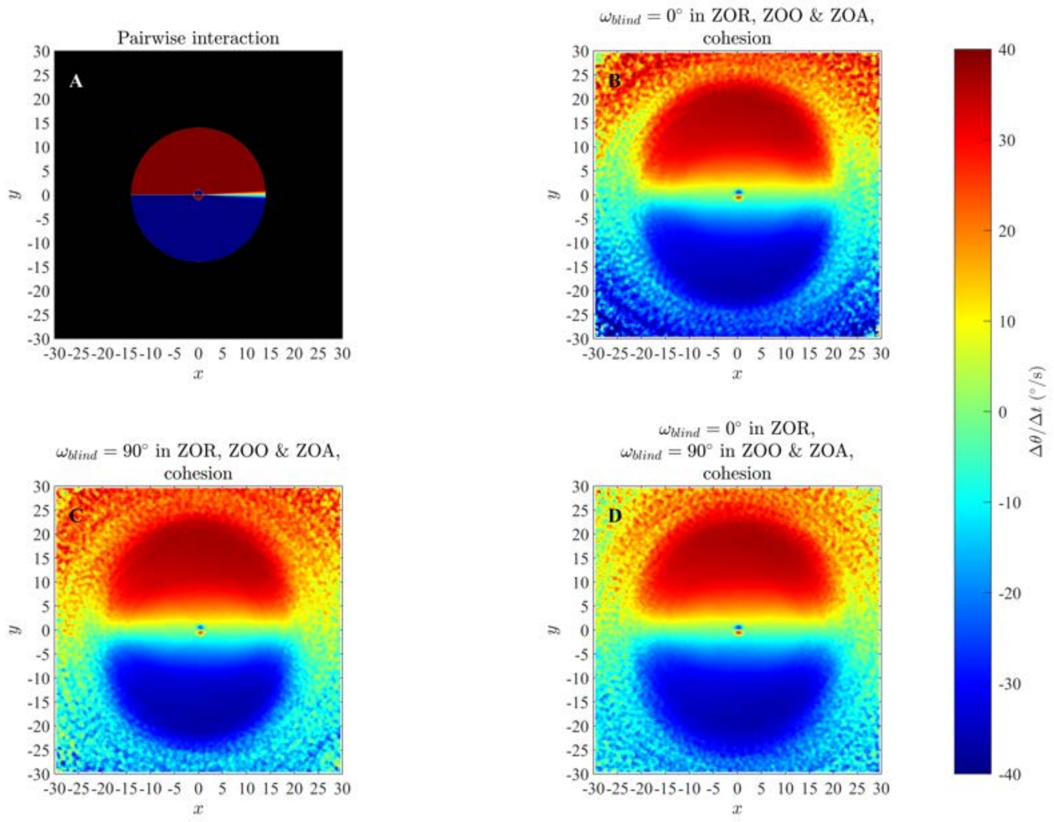


**Fig SF16.** Panel A: analytical pairwise interactions for given parameter values, as described in section S3.1, where turning of the individuals is governed by equations (S3.5) and (S3.10). Panels B, C and D illustrate changes in direction of motion of individuals as a function of the relative positions of partners obtained via analysis of simulations with $r_r = 1$, $\Delta r_o = 0.01$ and $\Delta r_a = 12.99$ using the averaging method. ($N = 25$, 10000 time steps per simulation.)

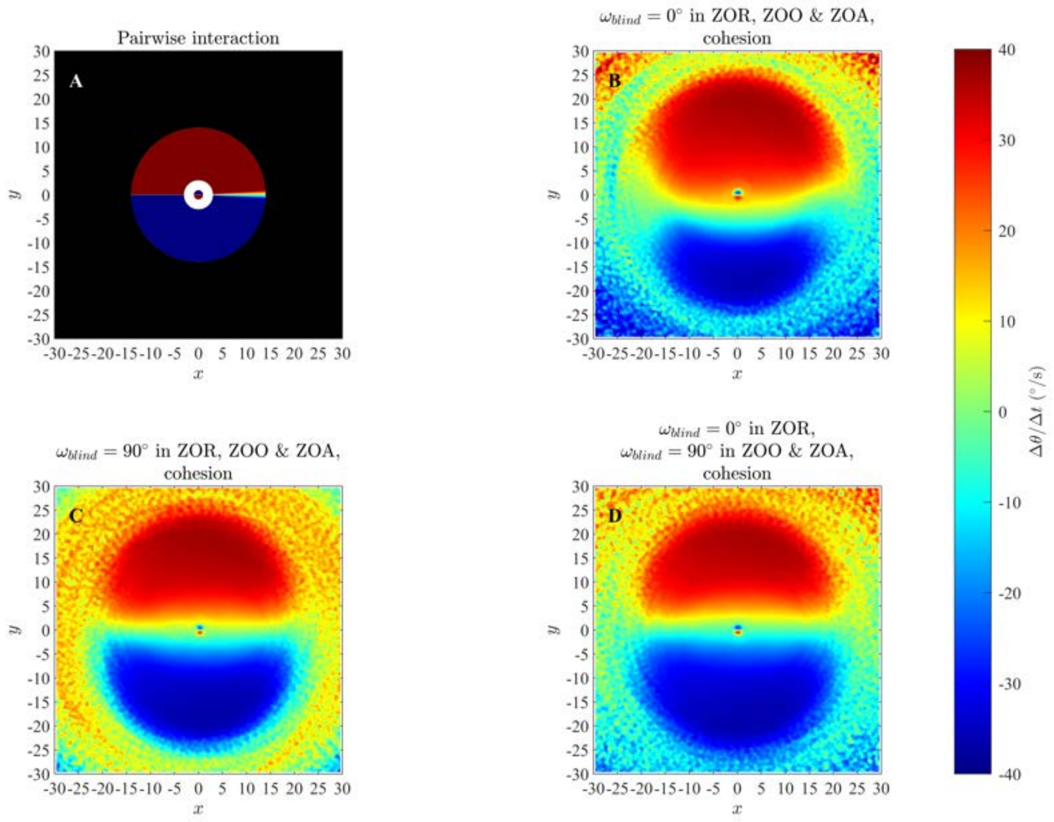


**Fig SF17.** Panel A: analytical pairwise interactions for given parameter values, as described in section S3.1, where turning of the individuals is governed by equations (S3.5) and (S3.10). Panels B, C and D illustrate changes in direction of motion of individuals as a function of the relative positions of partners obtained via analysis of simulations with $r_r = 1$, $\Delta r_o = 2$ and $\Delta r_a = 11$ using the averaging method. ($N = 25$, 10000 time steps per simulation.)

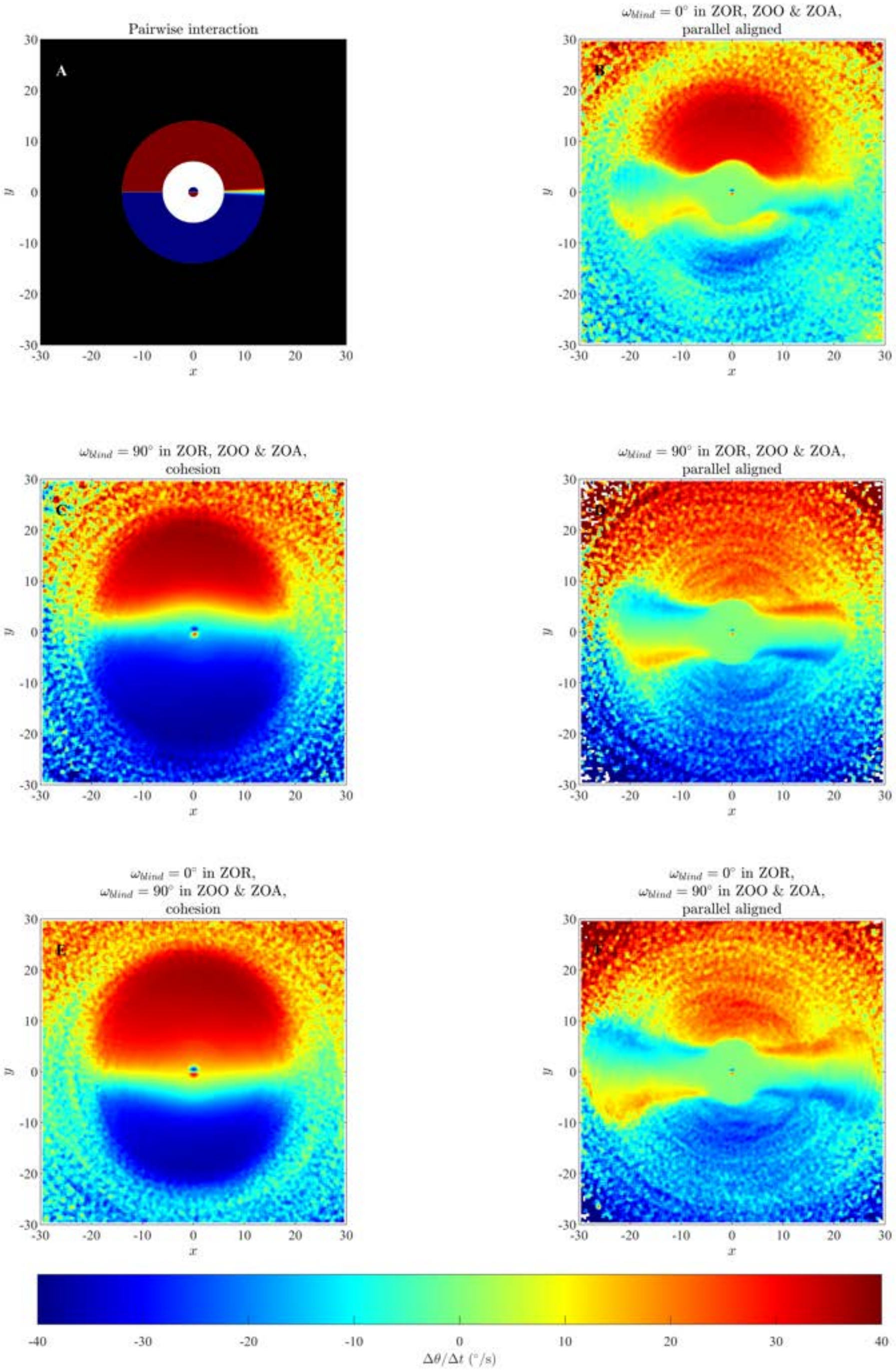


**Fig SF18.** Panel A: analytical pairwise interactions for given parameter values, as described in section S3.1, where turning of the individuals is governed by equations (S3.5) and (S3.10). Panels B, C, D, E and F illustrate changes in direction of motion of individuals as a function of the relative positions of partners obtained via analysis of simulations with $r_r = 1$, $\Delta r_o = 5$ and $\Delta r_a = 8$ using the averaging method. ($N = 25$, 10000 time steps per simulation.)

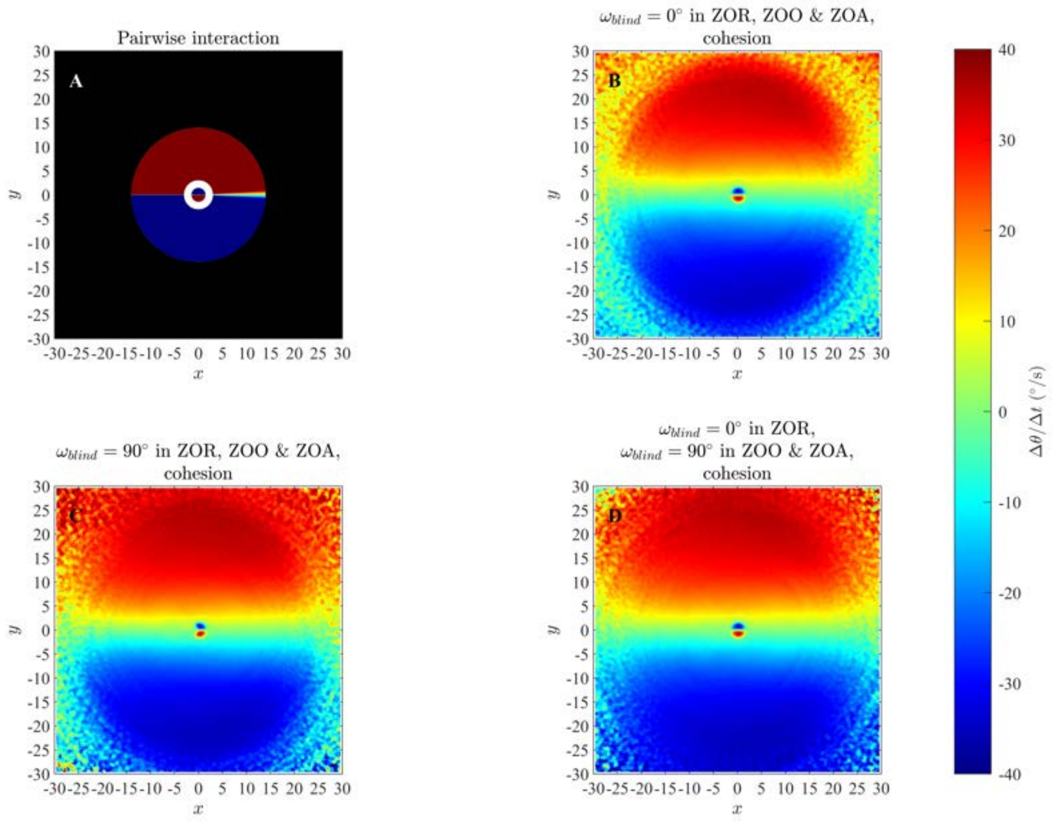


**Fig SF19.** Panel A: analytical pairwise interactions for given parameter values, as described in section S3.1, where turning of the individuals is governed by equations (S3.5) and (S3.10). Panels B, C and D illustrate changes in direction of motion of individuals as a function of the relative positions of partners obtained via analysis of simulations with $r_r = 1.5$, $\Delta r_o = 1.5$ and $\Delta r_a = 11$ using the averaging method. ($N = 25$, 10000 time steps per simulation.)

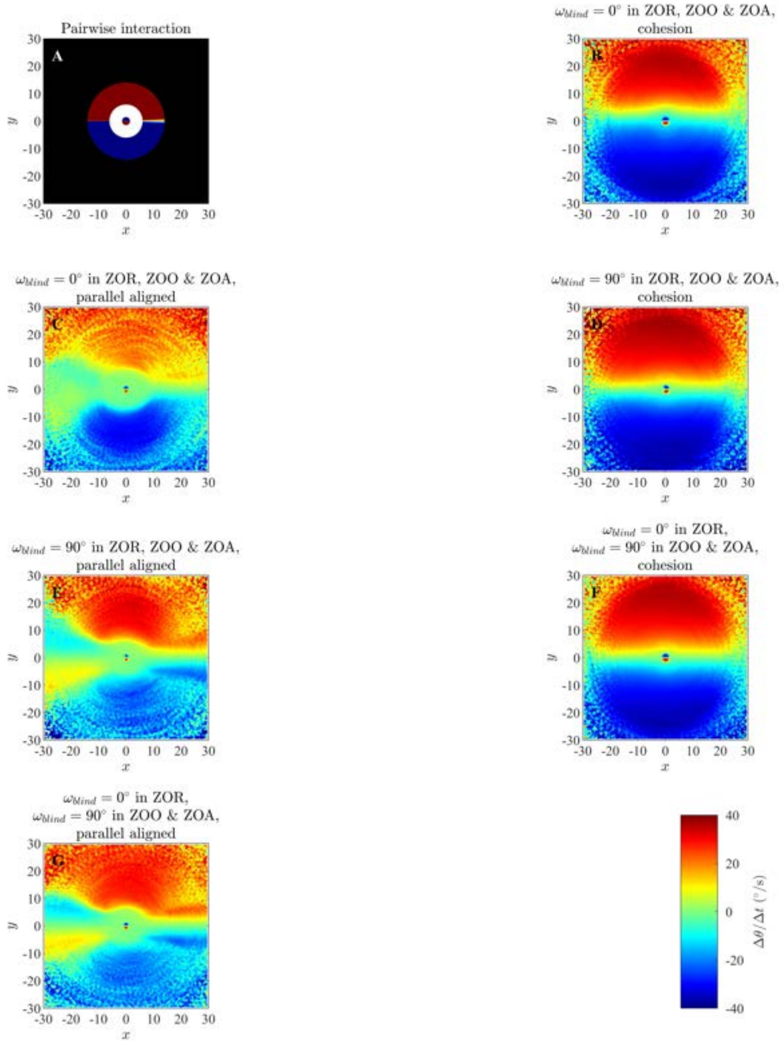


**Fig SF20.** Panel A: analytical pairwise interactions for given parameter values, as described in section S3.1, where turning of the individuals is governed by equations (S3.5) and (S3.10). Panels B, C, D, E, F and G illustrate changes in direction of motion of individuals as a function of the relative positions of partners obtained via analysis of simulations with $r_r = 1.5$, $\Delta r_o = 4.5$ and $\Delta r_a = 8$ using the averaging method. ($N = 25$, 10000 time steps per simulation.)

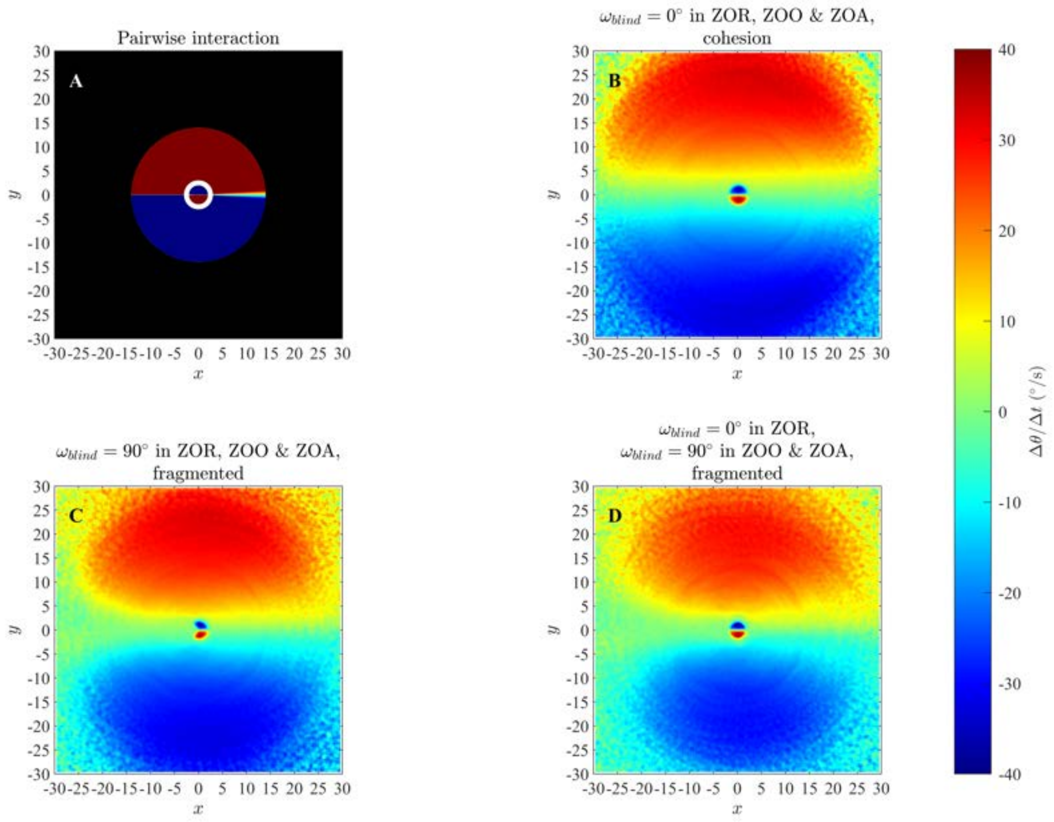


**Fig SF21.** Panel A: analytical pairwise interactions for given parameter values, as described in section S3.1, where turning of the individuals is governed by equations (S3.5) and (S3.10). Panels B, C and D illustrate changes in direction of motion of individuals as a function of the relative positions of partners obtained via analysis of simulations with $r_r = 2$, $\Delta r_o = 1$ and $\Delta r_a = 11$ using the averaging method. ($N = 25$, 10000 time steps per simulation.)

# S7 Group size effects on analysis of the zonal model

Figures 6 (in the main text) and SF22 illustrate the effects of group size on analysis of data derived from the zonal model.

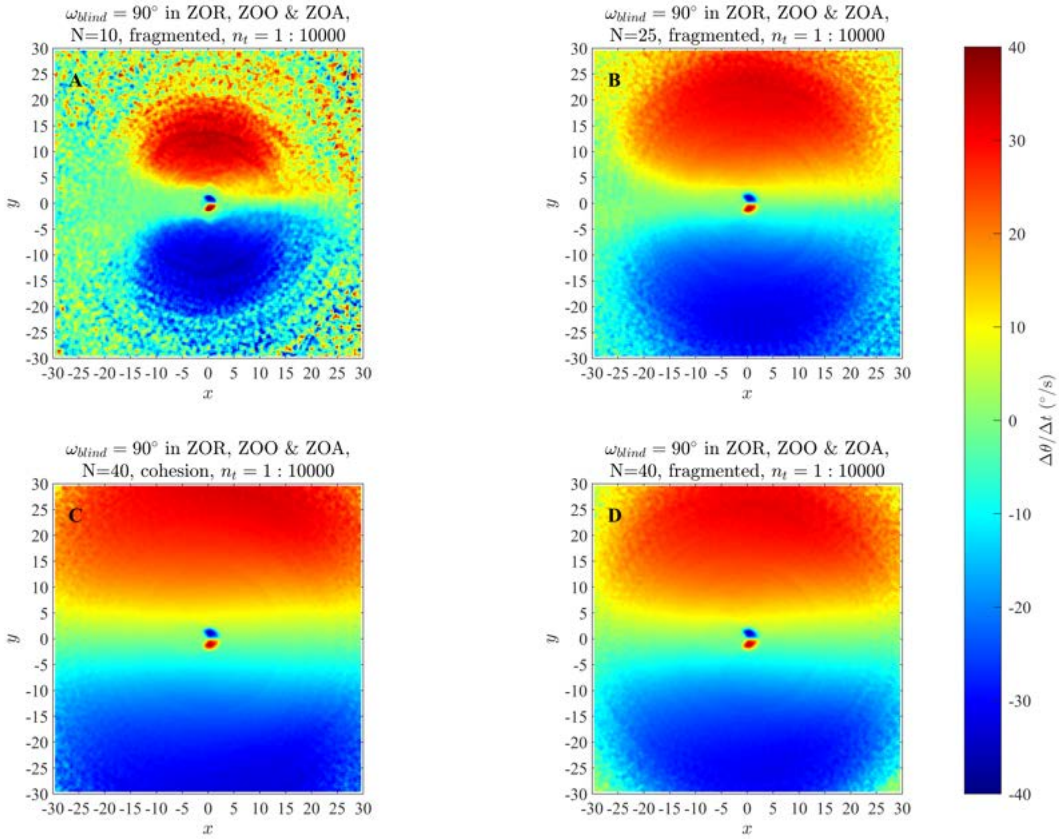


**Fig SF22.** Changes in direction of motion in groups of of $N = 10$, $N = 25$ and $N = 40$ individuals as a function of the relative positions of partners obtained via analysis of simulations with $r_r = 2$, $\Delta r_o = 1$, $\Delta r_a = 11$ and $n_t = 10000$ time steps using the averaging method.

# S8 Transient effects on analysis of the zonal model

Figures SF23 to SF25 illustrate the results of analysis applied to the first or second half of sets of 10000 time step simulations generated by the zonal model.

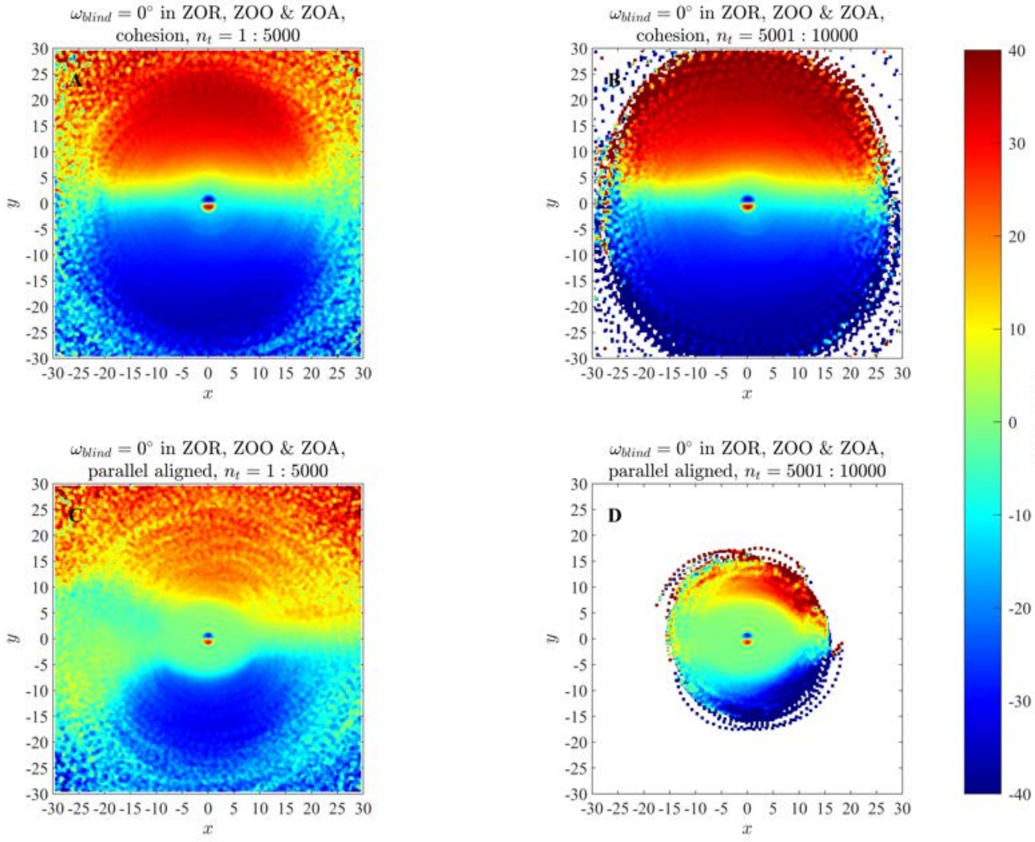


**Fig SF23.** Changes in direction of motion of individuals as a function of the relative positions of partners obtained via analysis of simulations with $r_r = 1.5$, $\Delta r_o = 4.5$ and $\Delta r_a = 8$ using the averaging method. Analysis was confined to the first 5000 time steps (left column), or final 5000 time steps (right column) of simulations run over 10000 time steps. Here $N = 25$.

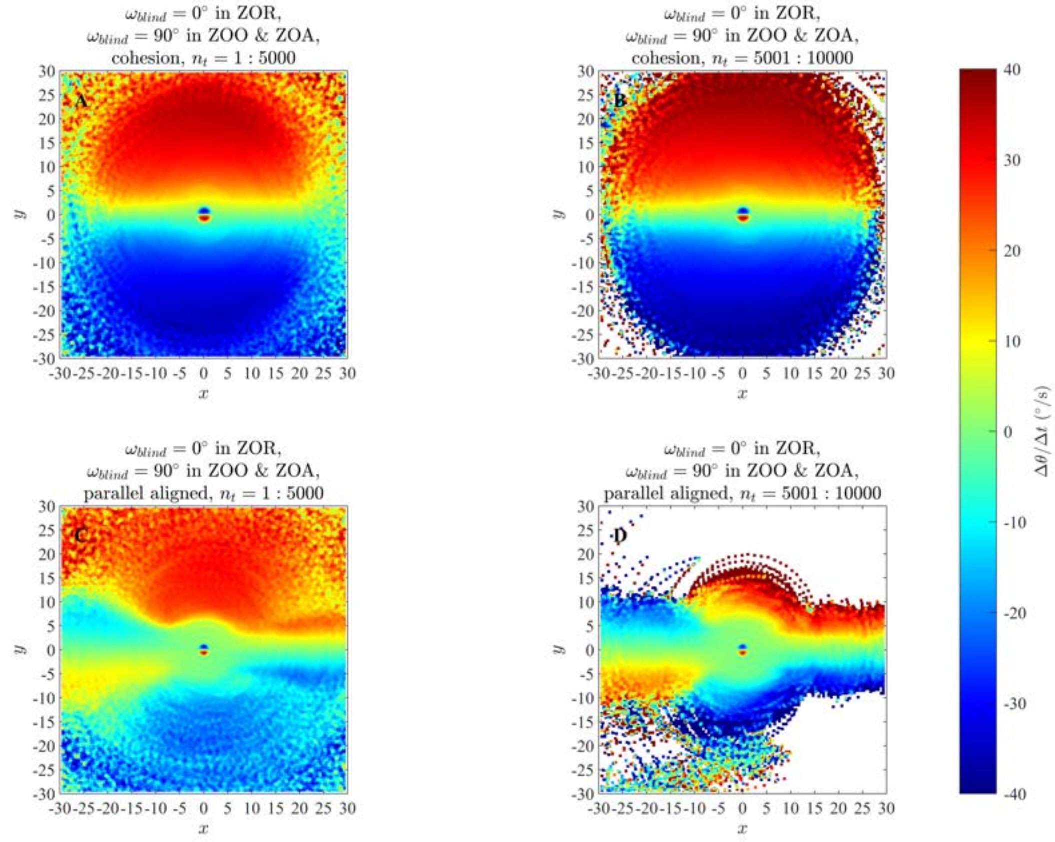


**Fig SF24.** Changes in direction of motion of individuals as a function of the relative positions of partners obtained via analysis of simulations with $r_r = 1.5$, $\Delta r_o = 4.5$ and $\Delta r_a = 8$ using the averaging method. Analysis was confined to the first 5000 time steps (left column), or final 5000 time steps (right column) of simulations run over 10000 time steps. Here $N = 25$.

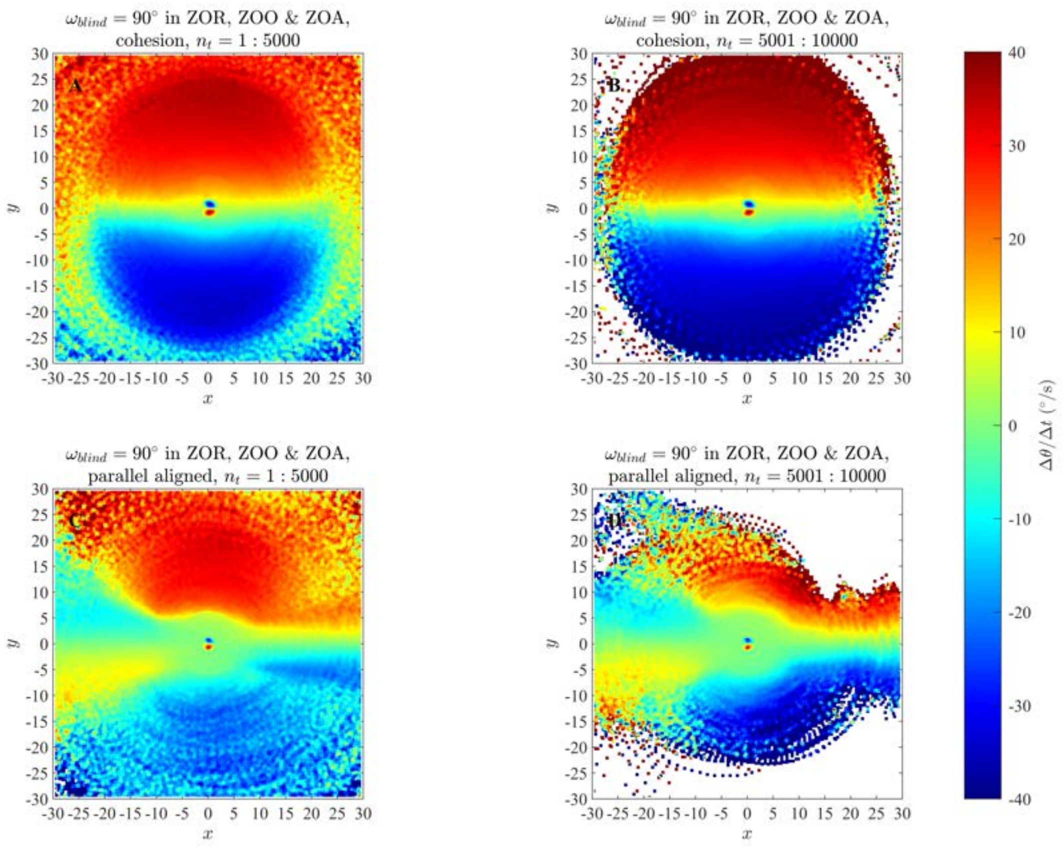


**Fig SF25.** Changes in direction of motion of individuals as a function of the relative positions of partners obtained via analysis of simulations with $r_r = 1.5$, $\Delta r_o = 4.5$ and $\Delta r_a = 8$ using the averaging method. Analysis was confined to the first 5000 time steps (left column), or final 5000 time steps (right column) of simulations run over 10000 time steps. Here $N = 25$.

# S9 Estimation of radius of ZOR and blind regions from zonal model simulations

## S9.1 Visual estimation of blind zone

To estimate the apparent blind angle from the graphs, we manualy selected three points $(x_1, y_1)$, $(x_2, y_2)$ and $(y_3, y_3)$ input by mouse clicks on the graph of the change in direction that were reasonable approximations to vertices of the triangle shape within the estimated ZOR. $(x_1, y_1)$ was selected near the center of the estimated ZOR and $(x_2, y_2)$ and $(x_3, y_3)$ were selected on the ZOR to enclose the v-shape. By means of example, the points $(x_1, y_1)$, $(x_2, y_2)$ and $(y_3, y_3)$ are represented as white asterisks on the graph in Figure 2. The angle

(representing the estimated blind angle) between the line segment joining the points $(x_1, y_1)$ and $(x_2, y_2)$ and the line segment joining the points $(x_1, y_1)$ and $(x_3, y_3)$ was calculated using the equation

$$\omega_{blind} = \cos^{-1}\left(\frac{(x_2 - x_1)(x_3 - x_1) + (y_2 - y_1)(y_3 - y_1)}{\sqrt{(x_2 - x_1)^2 + (y_2 - y_1)^2}\sqrt{(x_3 - x_1)^2 + (y_3 - y_1)^2}}\right). \tag{S9.1}$$

## S9.2 Supporting graphs

We planned to use Figures SF27 to SF31 to estimate the extent of repulsion zones and blind regions where such regions were easily distinguished, or the region over which repulsion applied was approximately circular. Figures SF26, SF27, and SF28 A were ultimately excluded from this analysis, as the repulsion regions in those graphs may have not been well approximated via circles, and/or the wedge shape in the blind zone was not clear.

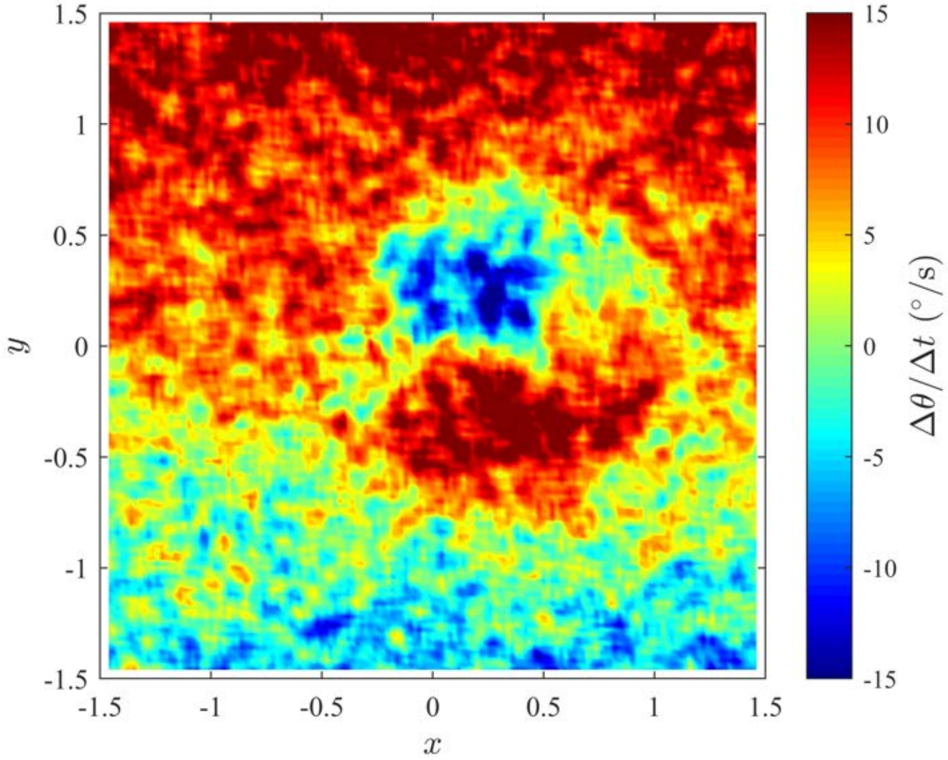


**Fig SF26.** Plot corresponding to Table 2: blind angle $\omega_{blind} = 90°$.
$r_r = 0.5; \Delta r_o = 0.51; \Delta r_a = 12.99$; cohesion. The focal individual is located at the origin moving right parallel to the $x$-axis. ($N = 25$ individuals.)

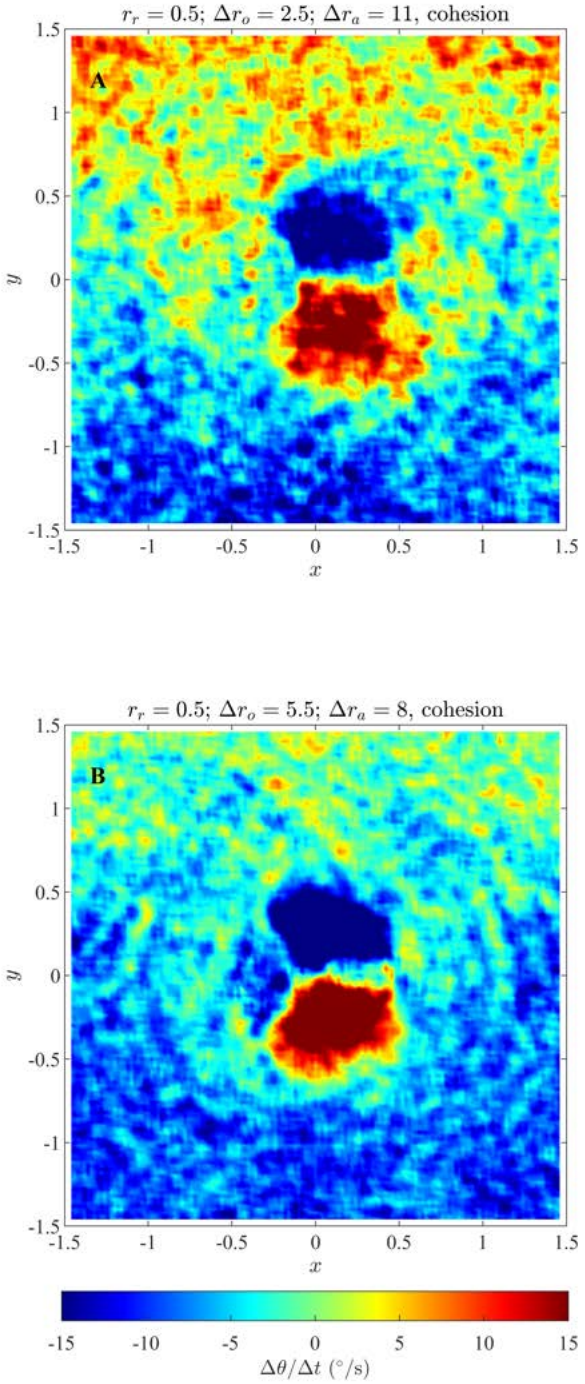


**Fig SF27.** Plot corresponding to Table 2: blind angle $\omega_{blind} = 90°$. The focal individual is located at the origin moving right parallel to the $x$-axis. ($N = 25$ individuals.)

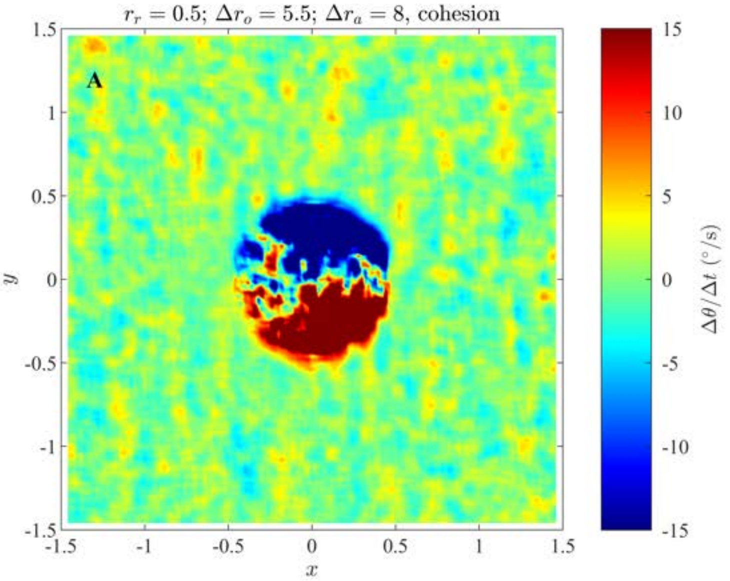


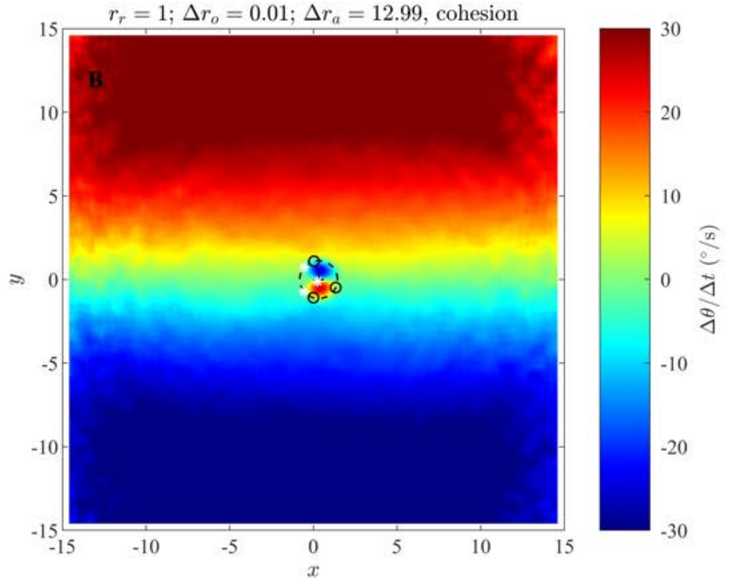


**Fig SF28.** Plot corresponding to Table 2: blind angle $\omega_{blind} = 90°$. The focal individual is located at the origin moving right parallel to the $x$-axis. Small black circle represents the points that are used to estimate the circle bounding the ZOR. The white asterisks represents the points used to estimate the size of blind angle. ($N = 25$ individuals.)

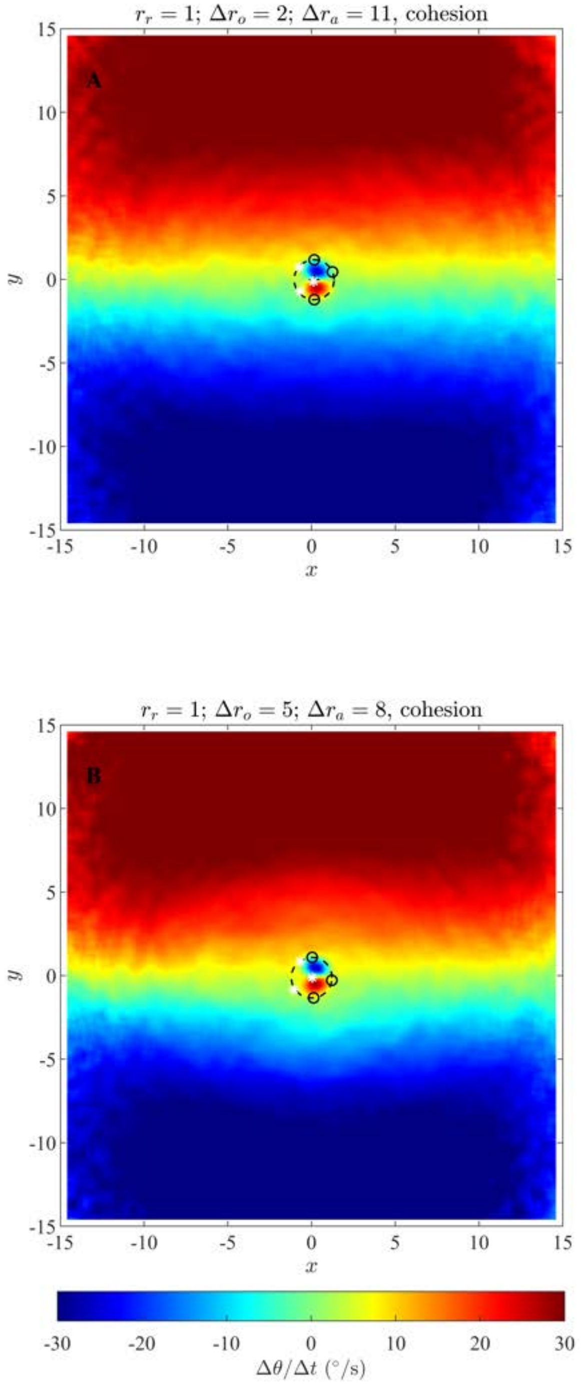


**Fig SF29.** Plot corresponding to Table 2: blind angle $\omega_{blind} = 90°$. The focal individual is located at the origin moving right parallel to the $x$-axis. Small black circle represents the points that are used to estimate the circle bounding the ZOR. The white asterisks represents the points used to estimate the size of blind angle. ($N = 25$ individuals.)

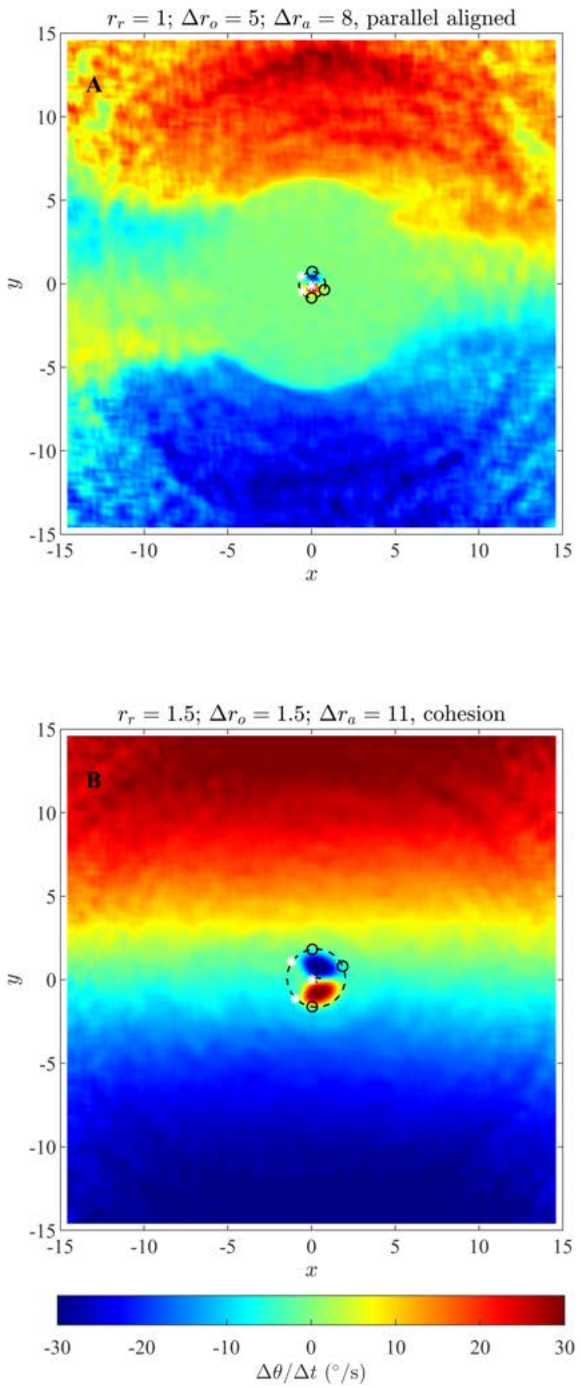


**Fig SF30.** Plot corresponding to Table 2: blind angle $\omega_{blind} = 90°$. The focal individual is located at the origin moving right parallel to the $x$-axis. Small black circle represents the points that are used to estimate the circle bounding the ZOR. The white asterisks represents the points used to estimate the size of blind angle. ($N = 25$ individuals.)

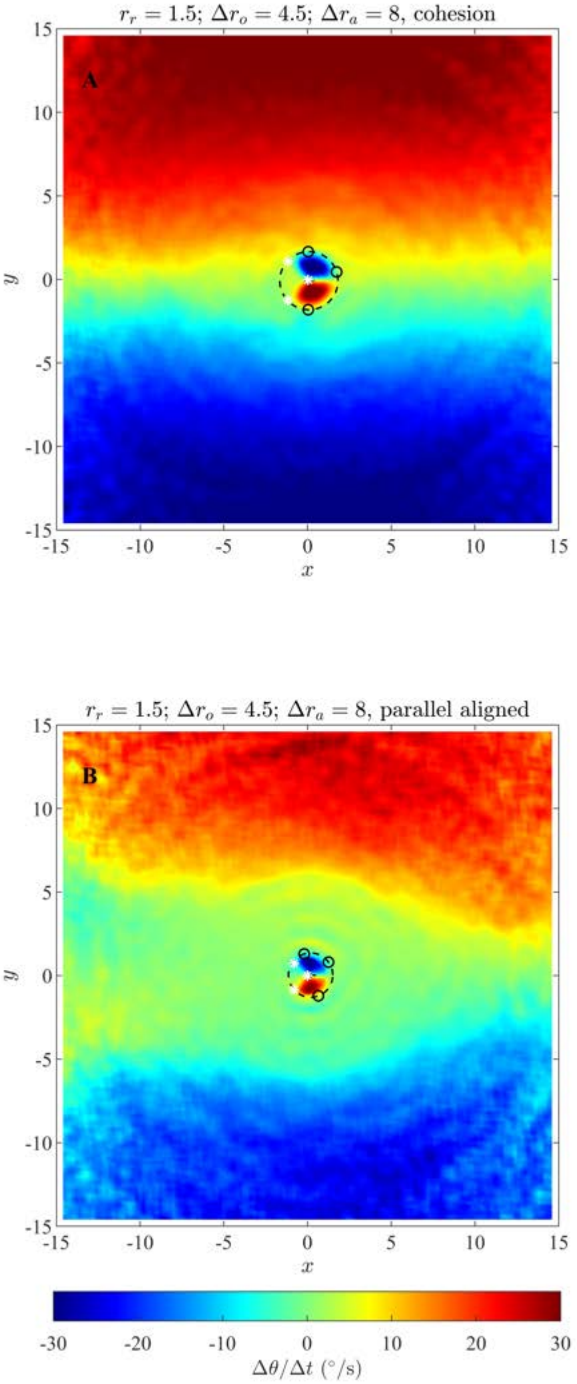


**Fig SF31.** Plot corresponding to Table 2: blind angle $\omega_{blind} = 90°$. The focal individual is located at the origin moving right parallel to the $x$-axis. Small black circle represents the points that are used to estimate the circle bounding the ZOR. The white asterisks represents the points used to estimate the blind angle. ($N = 25$ individuals.)

# S10 Supplementary plots derived from the ODE model with $N = 10$ individuals

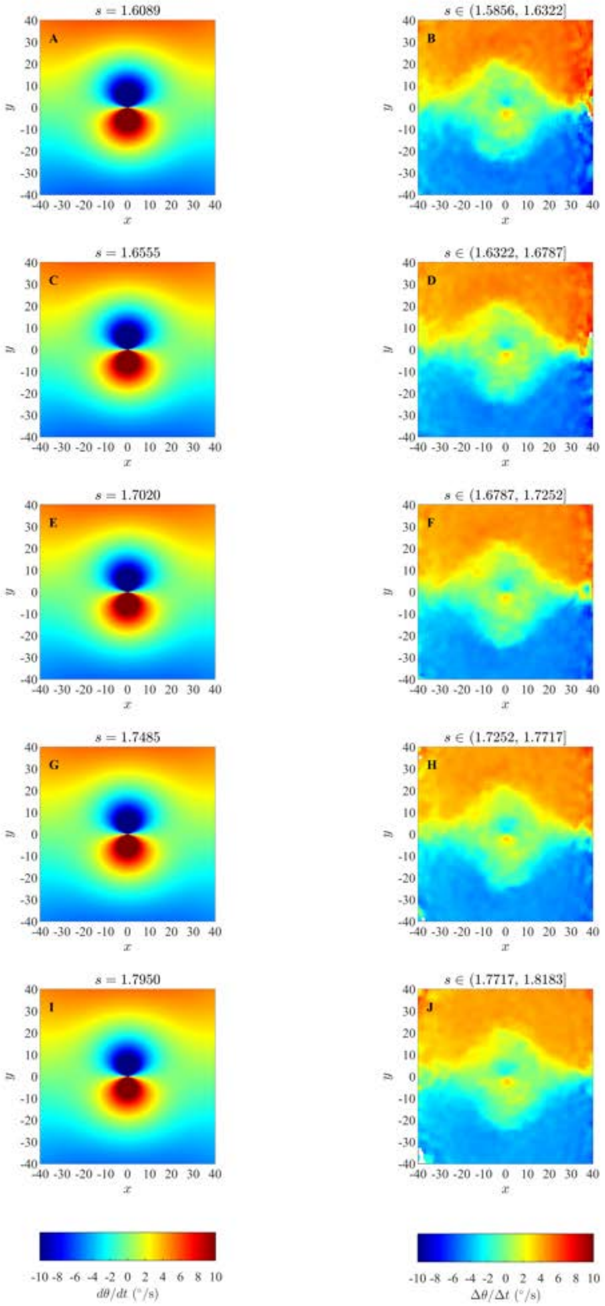


**Fig SF32.** Left column: analytical pairwise turning interactions as prescribed by the ODE model (equation (S3.19)) for groups that form double mills (item (a) from Table 3).

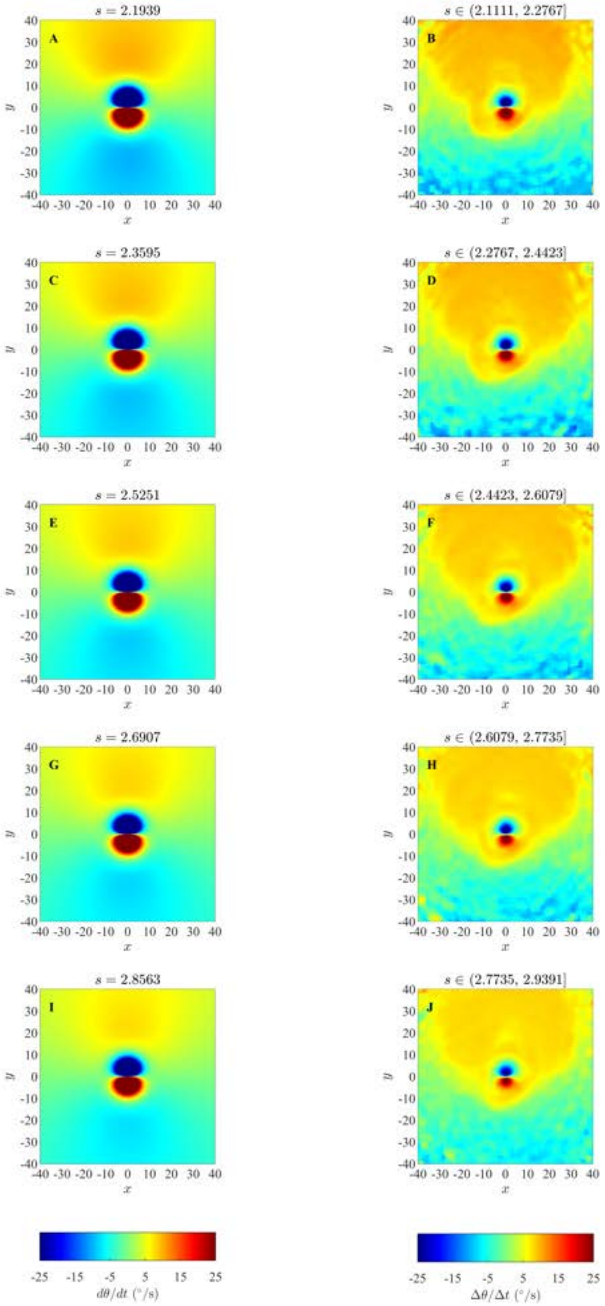


**Fig SF33.** Left column: analytical pairwise turning interactions as prescribed by the ODE model (equation (S3.19)) for groups that form anticlockwise rotating mills (item (b) from Table 3). Right column: results obtained via the averaging method.

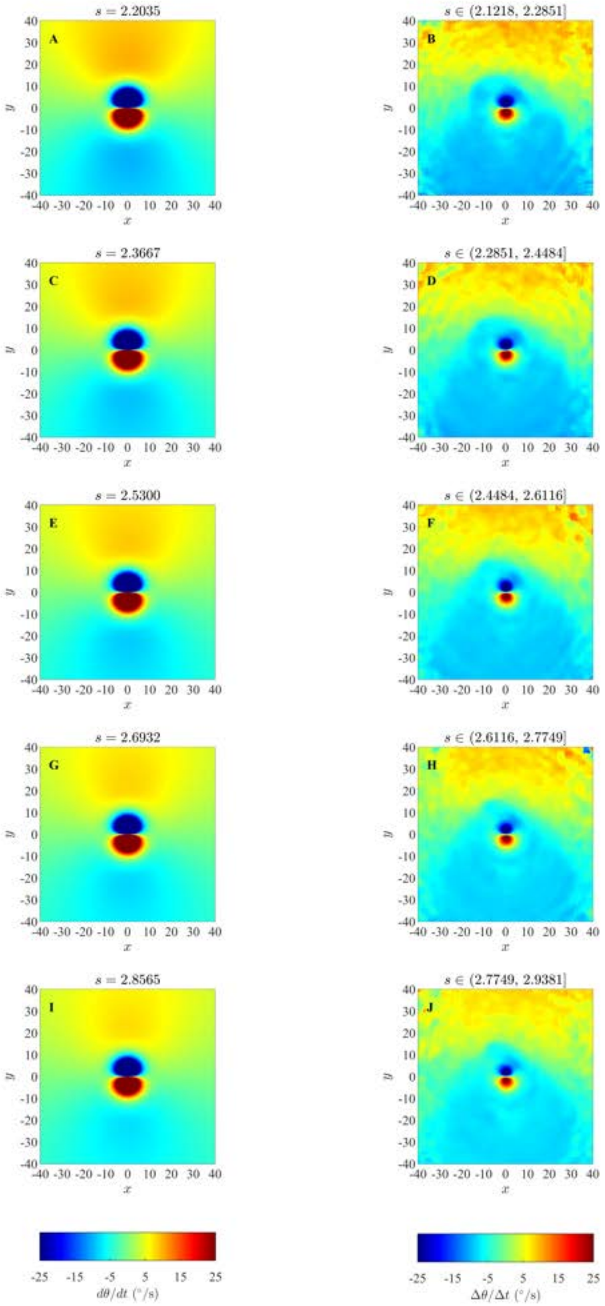

**Fig SF34.** Left column: analytical pairwise turning interactions as prescribed by the ODE model (equation (S3.19)) for groups that form clockwise rotating mills (item (b) from Table 3). Right column: results obtained via the averaging method.

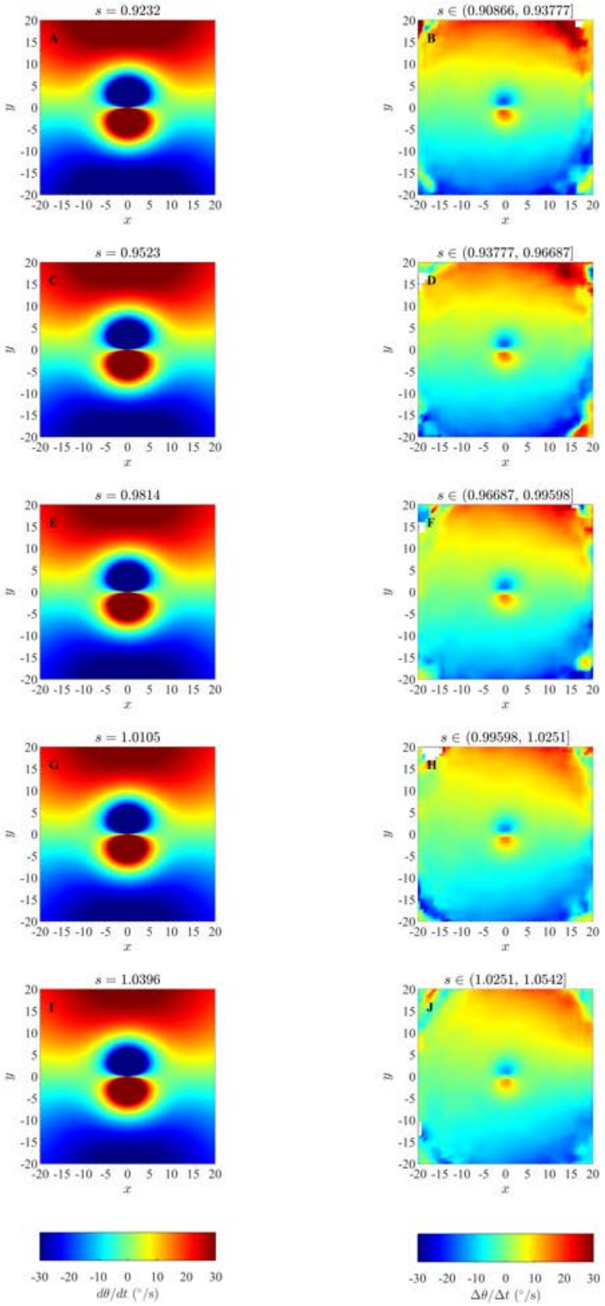


**Fig SF35.** Left column: analytical pairwise turning interactions as prescribed by the ODE model (equation (S3.19)) for groups that form swarms (item (c) from Table 3). Right column: results obtained via the averaging method.

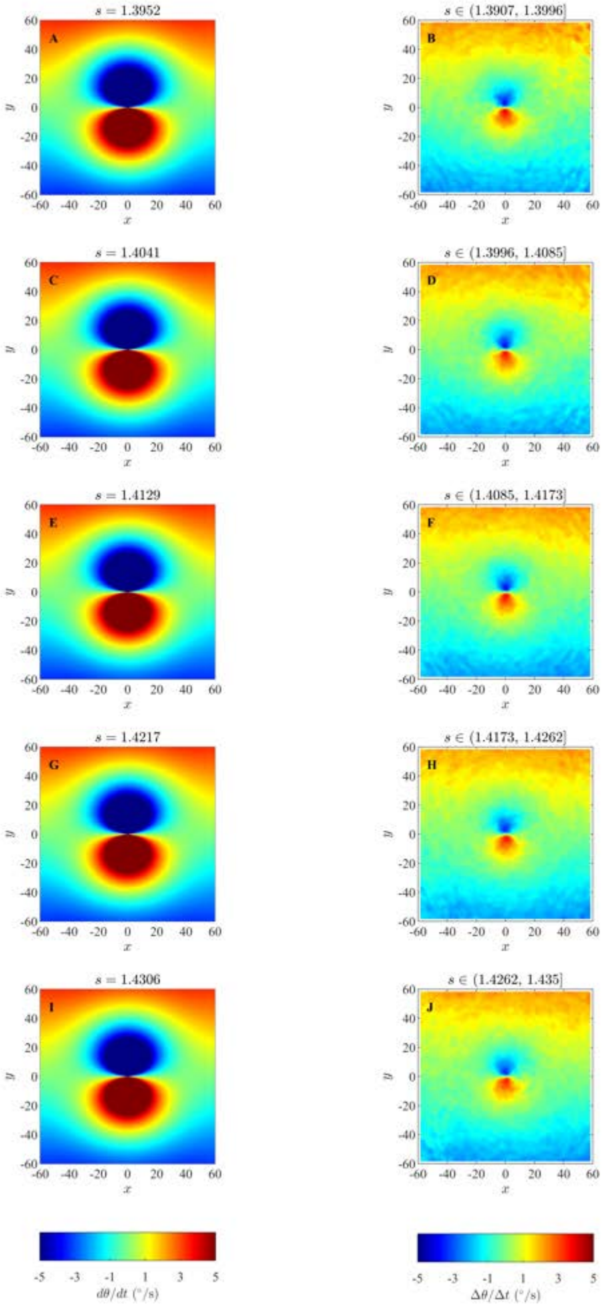


**Fig SF36.** Left column: analytical pairwise turning interactions as prescribed by the ODE model (equation (S3.19)) for groups that form swarms (item (d) from Table 3). Right column: results obtained via the averaging method.

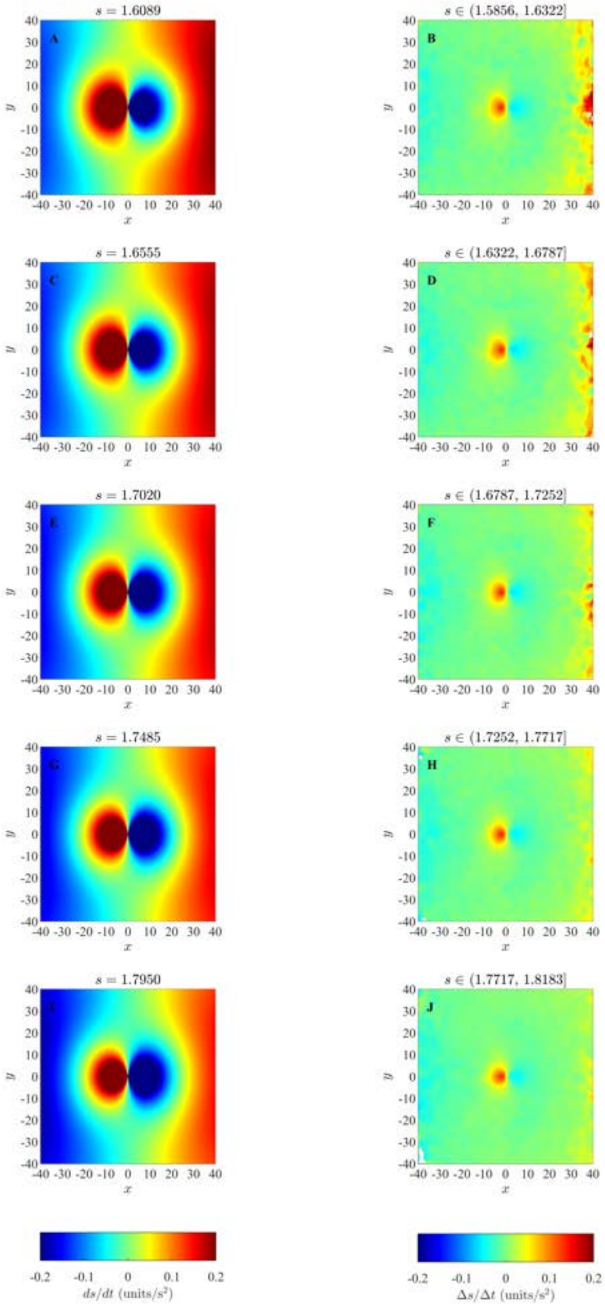


**Fig SF37.** Left column: analytical pairwise changes in speed as prescribed by the ODE model (equation (S3.18)) for groups that form double mills (item (a) from Table 3).

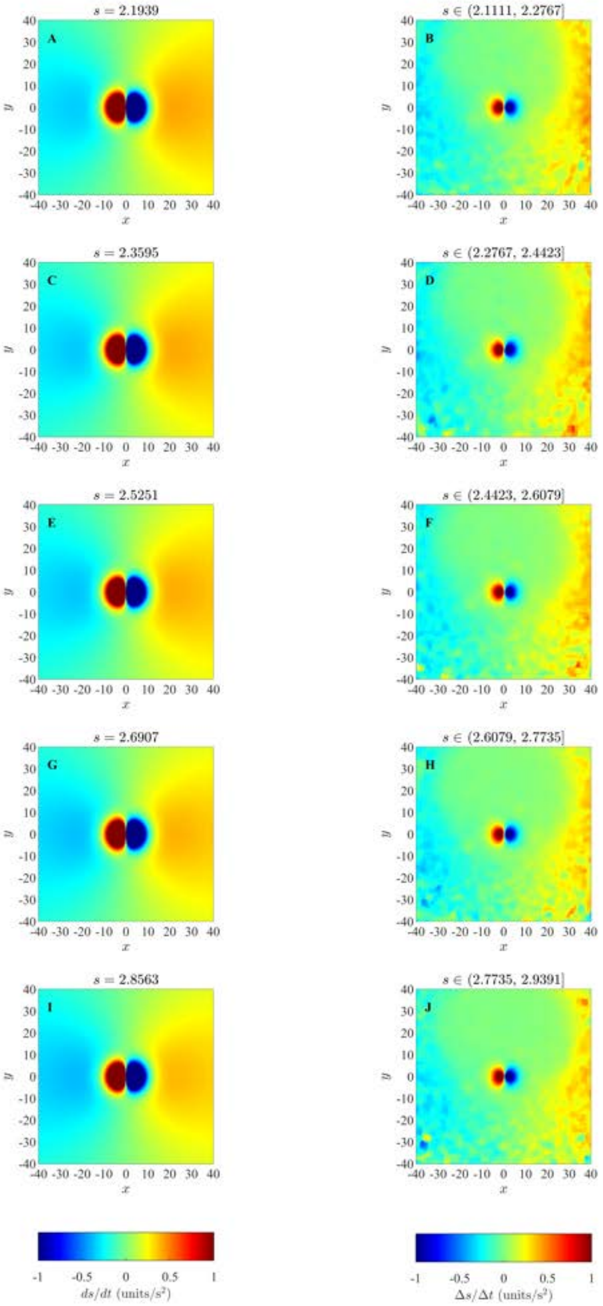


**Fig SF38.** Left column: analytical pairwise changes in speed as prescribed by the ODE model (equation (S3.18)) for groups that form anticlockwise rotating mills (item (b) from Table 3). Right column: results obtained via the averaging method.

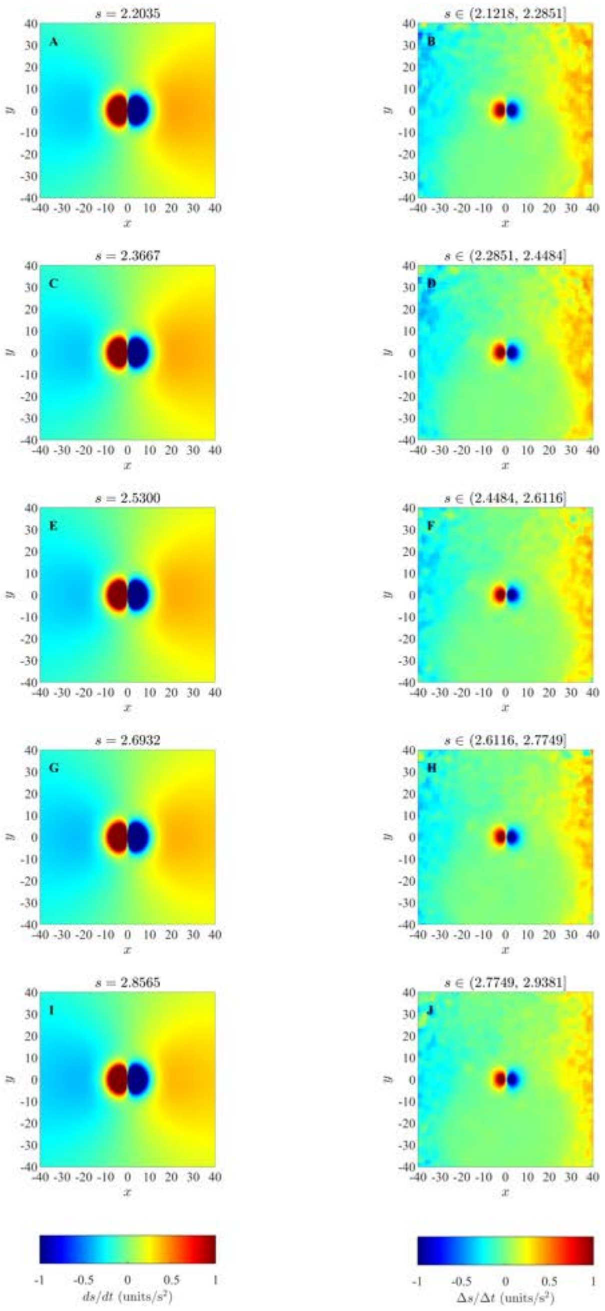


**Fig SF39.** Left column: analytical pairwise changes in speed as prescribed by the ODE model (equation (S3.18)) for groups that form clockwise rotating mills (item (b) from Table 3). Right column: results obtained via the averaging method.

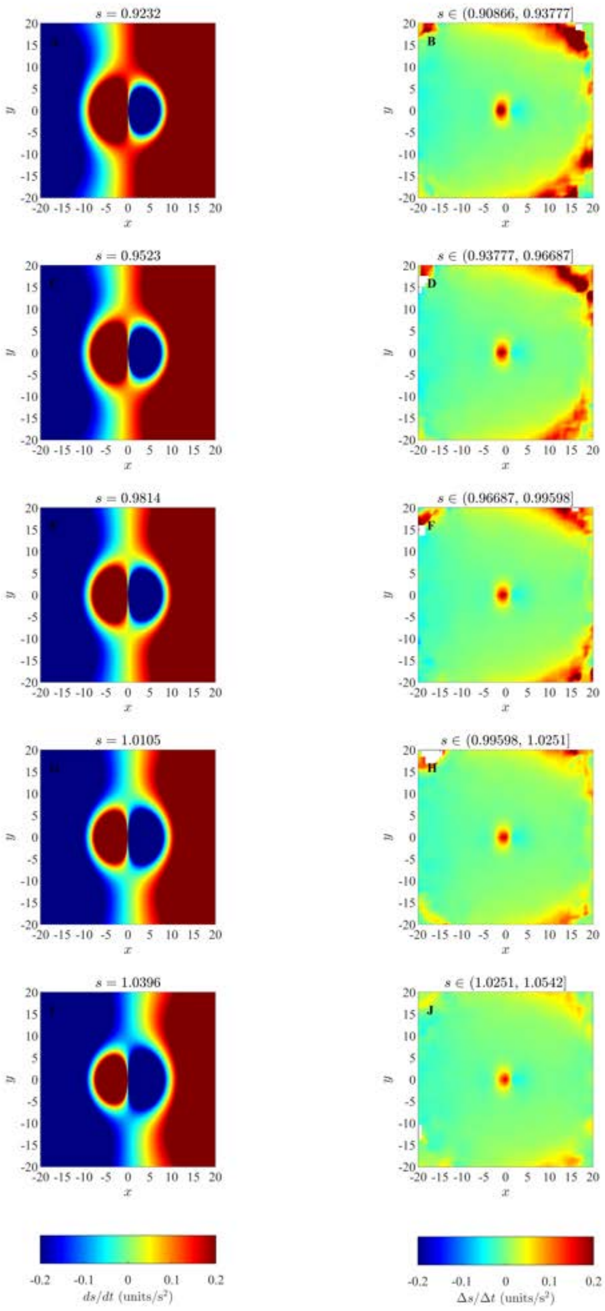

**Fig SF40.** Left column: analytical pairwise changes in speed as prescribed by the ODE model (equation (S3.18)) for groups that form swarms (item (c) from Table 3). Right column: results obtained via the averaging method.

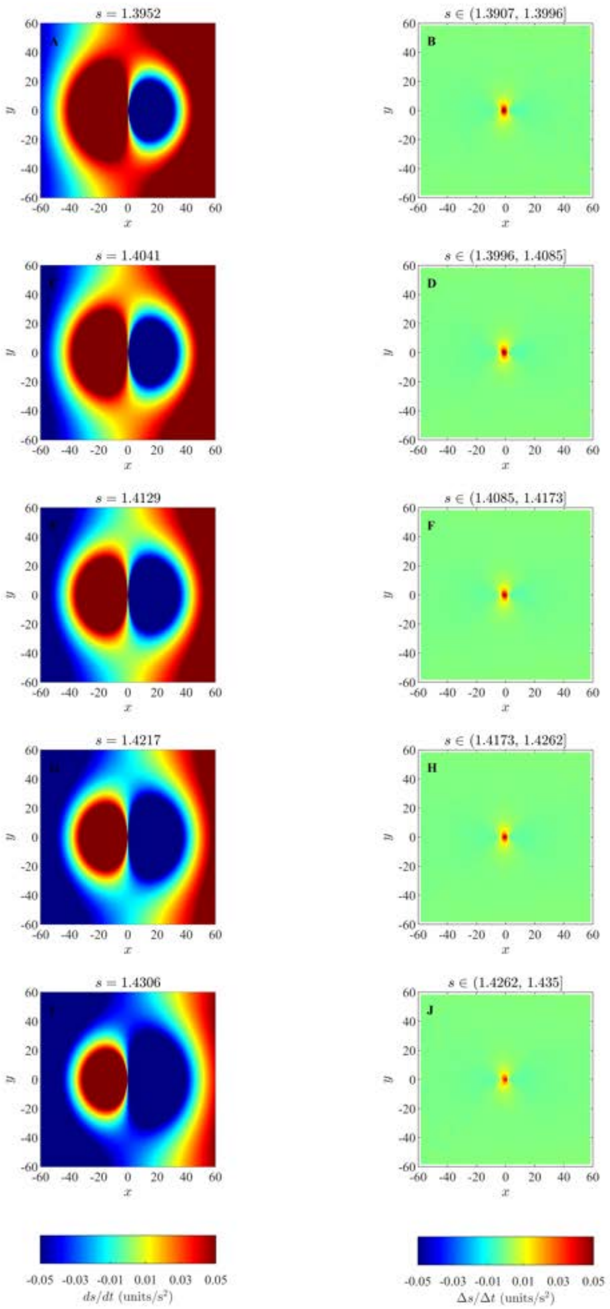


**Fig SF41.** Left column: analytical pairwise changes in speed as prescribed by the ODE model (equation (S3.18)) for groups that form swarms (item (d) from Table 3). Right column: results obtained via the averaging method.

# S11 Group size effects on analysis of the ODE model

Figures SF42 to SF51 illustrate the effects of varying group size on the results of analysis of trajectories from the ODE model.

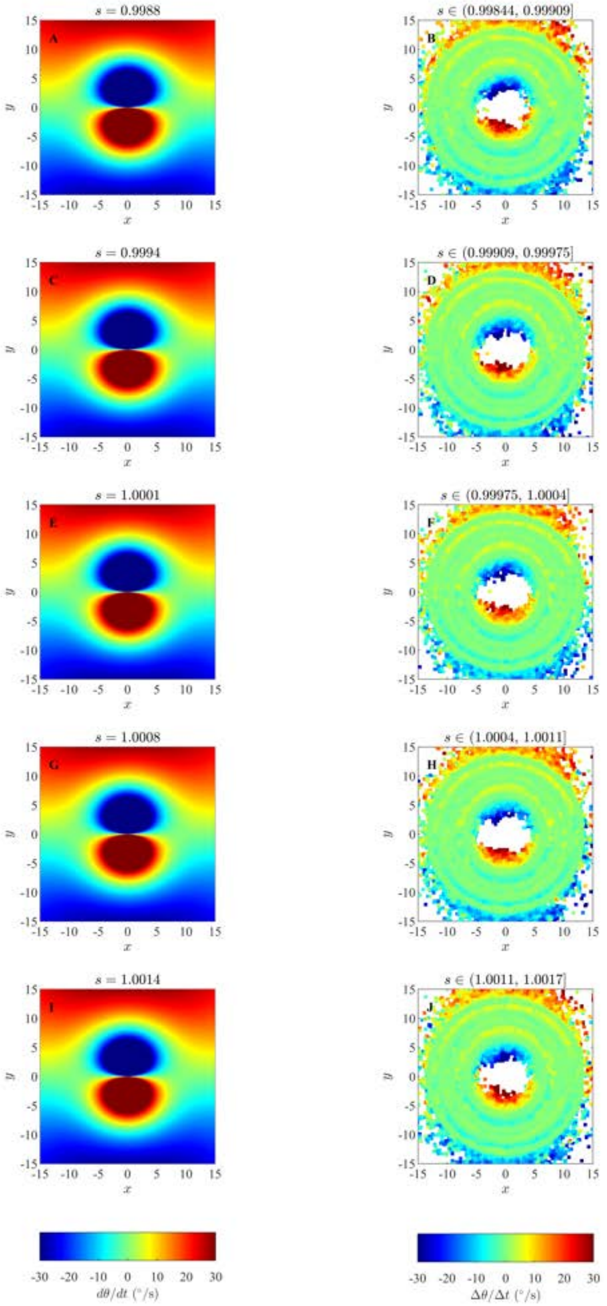


**Fig SF42.** Left column: analytical pairwise turning interactions as prescribed by the ODE model (equation (S3.19)) for groups for groups that undergo parallel motion (item (c) from Table 3, N=5). Right column: results obtained via the averaging method.

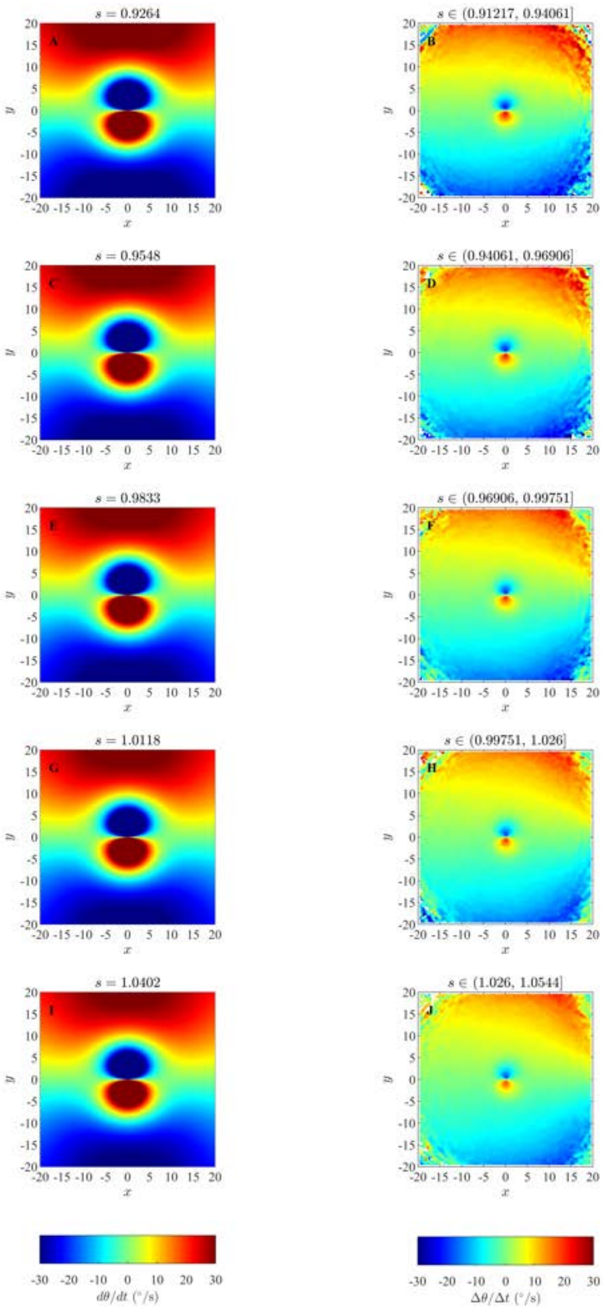


**Fig SF43.** Left column: analytical pairwise turning interactions as prescribed by the ODE model (equation (S3.19)) for groups that form swarms (item (c) from Table 3, N=15). Right column: results obtained via the averaging method.

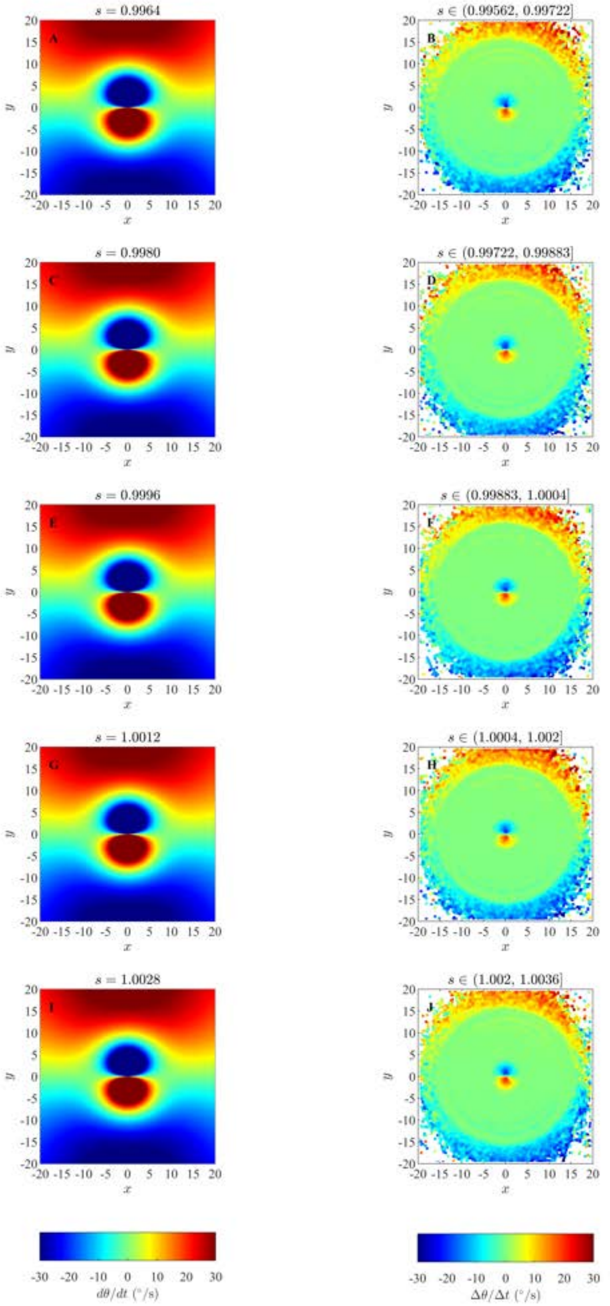


**Fig SF44.** Left column: analytical pairwise turning interactions as prescribed by the ODE model (equation (S3.19)) for groups for groups that undergo parallel motion (item (c) from Table 3, N=15). Right column: results obtained via the averaging method.

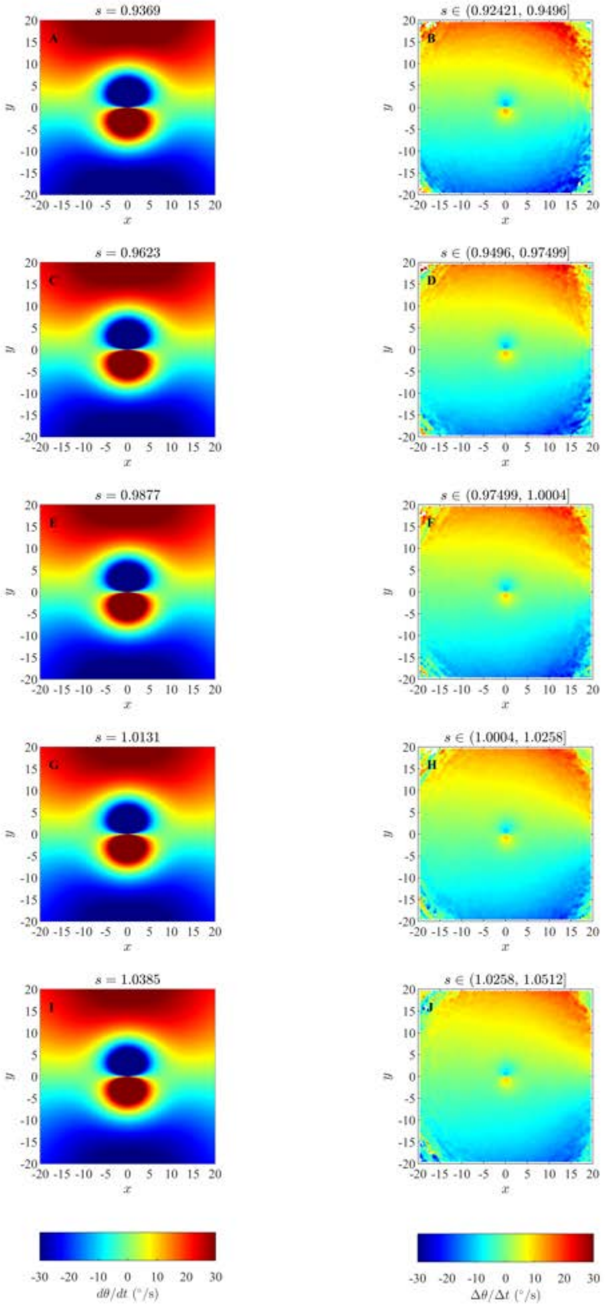


**Fig SF45.** Left column: analytical pairwise turning interactions as prescribed by the ODE model (equation (S3.19)) for groups that form swarms (item (c) from Table 3, N=25). Right column: results obtained via the averaging method.

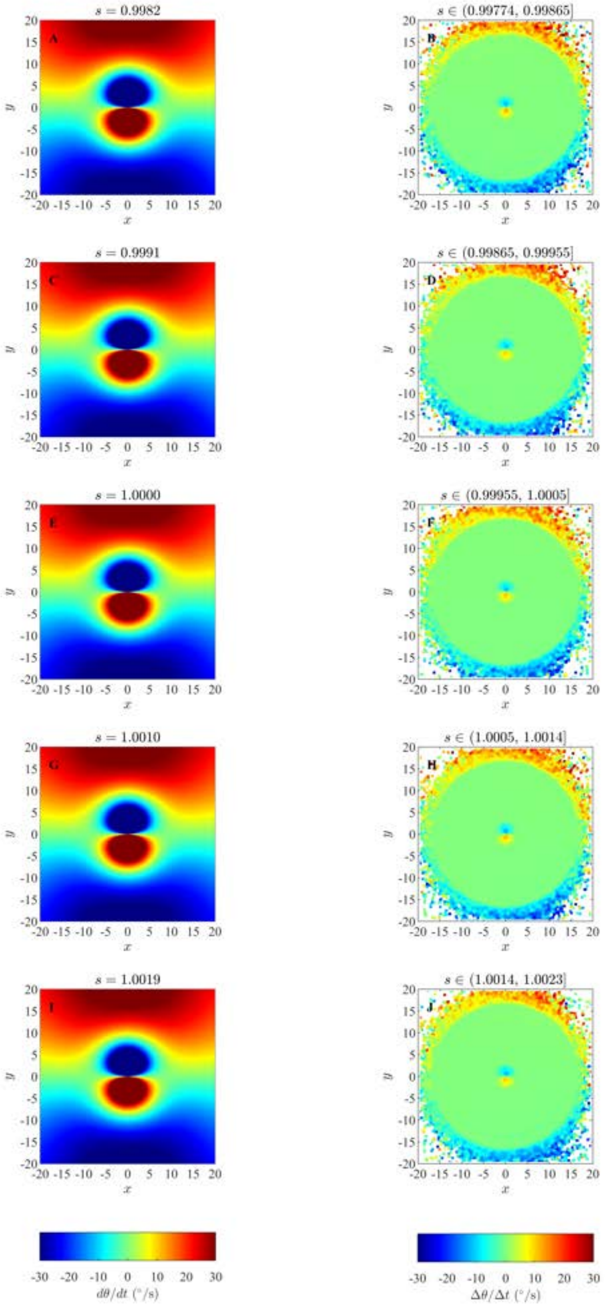


**Fig SF46.** Left column: analytical pairwise turning interactions as prescribed by the ODE model (equation (S3.19)) for groups for groups that undergo parallel motion (item (c) from Table 3, N=25). Right column: results obtained via the averaging method.

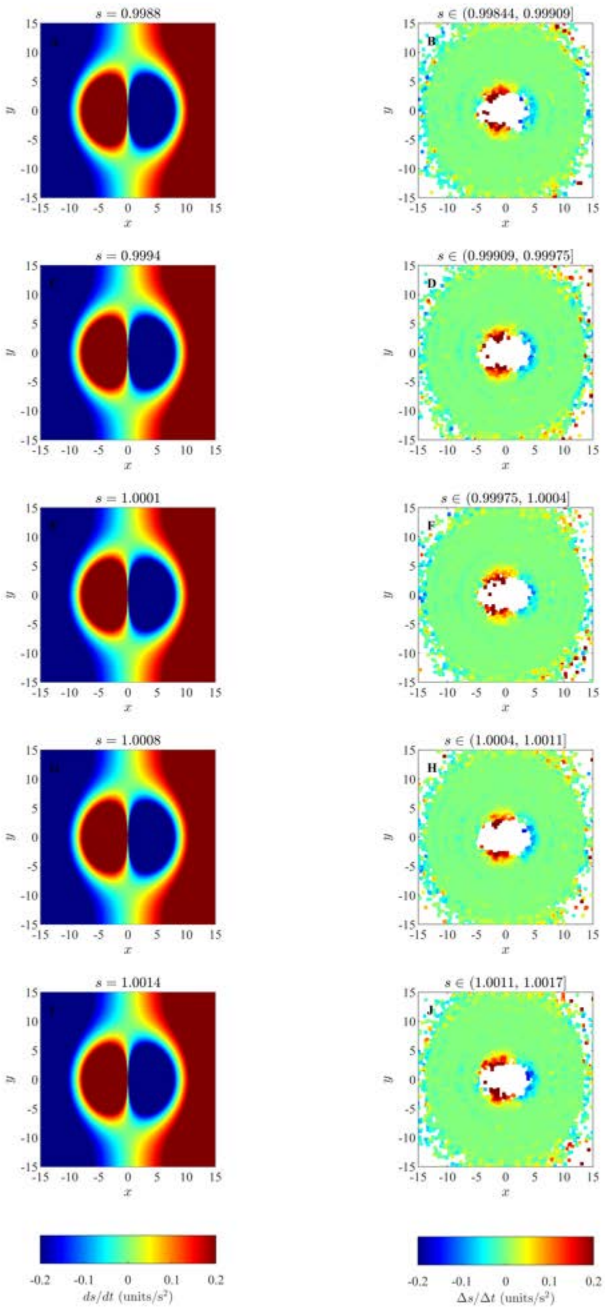


**Fig SF47.** Left column: analytical pairwise changes in speed as prescribed by the ODE model (equation (S3.18)) for groups that undergo parallel motion (item (c) from Table 3, N=5). Right column: results obtained via the averaging method.

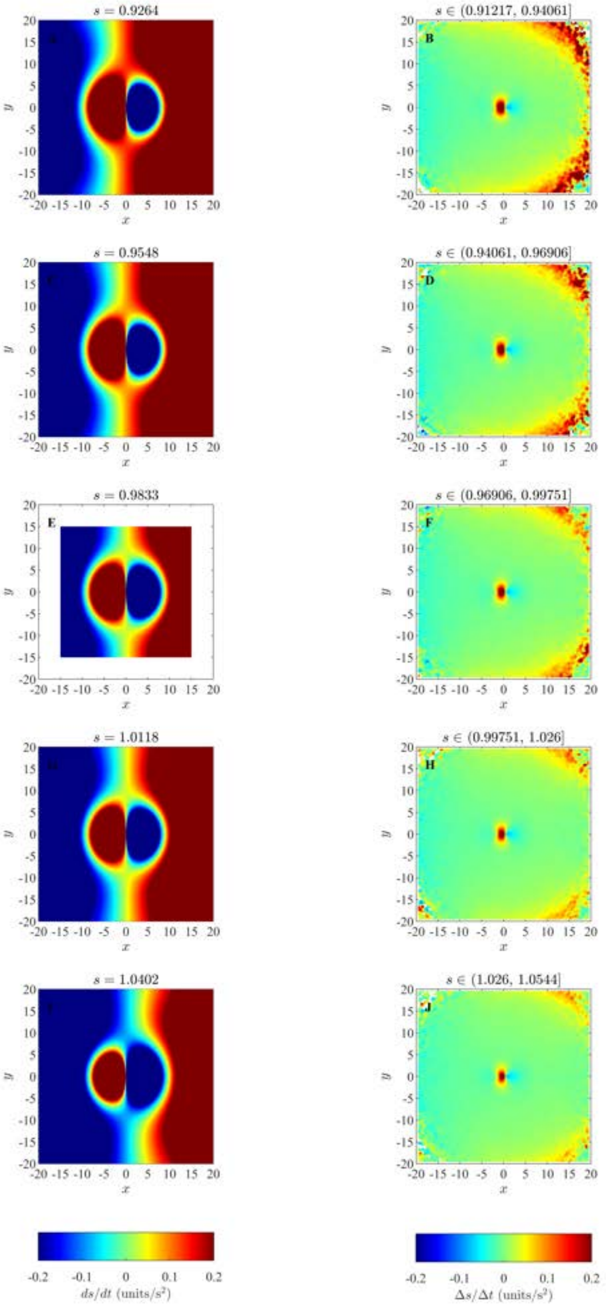


**Fig SF48.** Left column: analytical pairwise changes in speed as prescribed by the ODE model (equation (S3.18)) for groups that form swarms (item (c) from Table 3, N=15). Right column: results obtained via the averaging method.

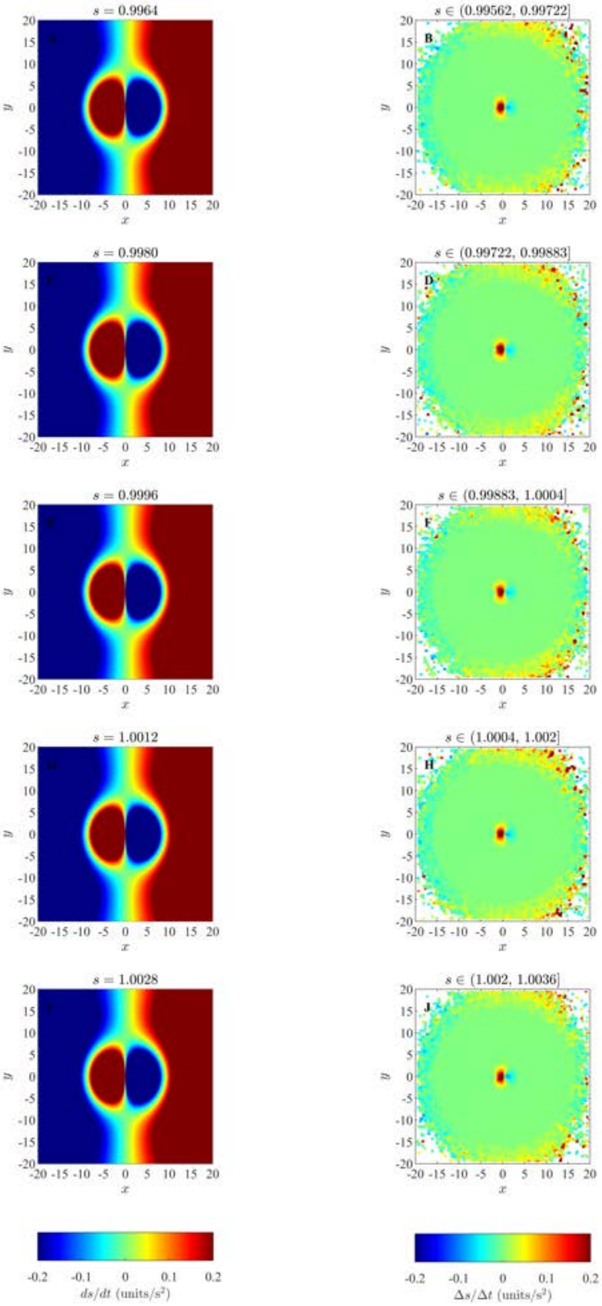

**Fig SF49.** Left column: analytical pairwise changes in speed as prescribed by the ODE model (equation (S3.18)) for groups that undergo parallel motion (item (c) from Table 3, N=15). Right column: results obtained via the averaging method.

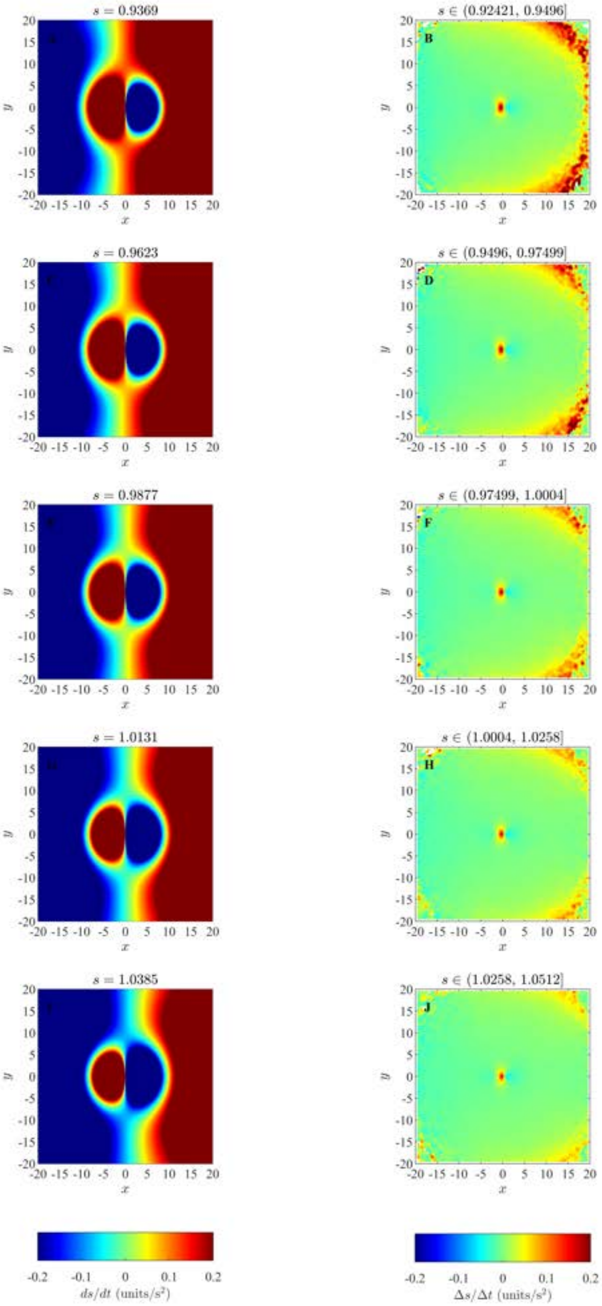


**Fig SF50.** Left column: analytical pairwise changes in speed as prescribed by the ODE model (equation (S3.18)) for groups that form swarms (item (c) from Table 3, N=25). Right column: results obtained via the averaging method.

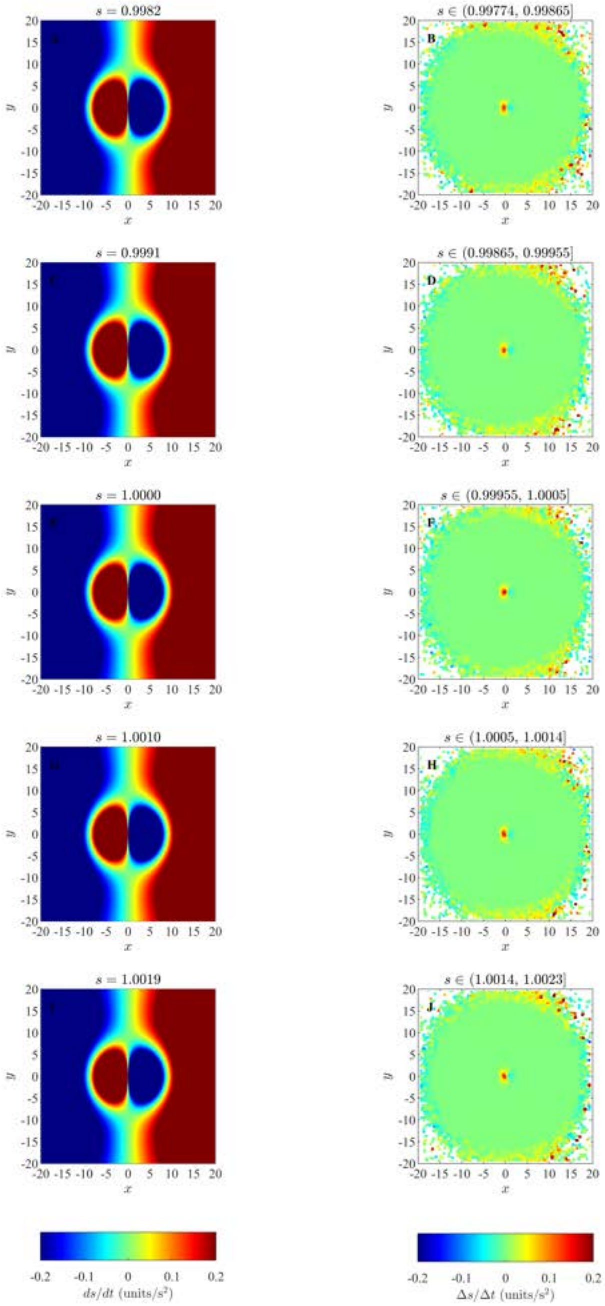


**Fig SF51.** Left column: analytical pairwise changes in speed as prescribed by the ODE model (equation (S3.18)) for groups that undergo parallel motion (item (c) from Table 3, N=25). Right column: results obtained via the averaging method.

# References

1. D'Orsogna MR, Chuang YL, Bertozzi AL, Chayes LS. Self-propelled particles with soft-core interactions: patterns, stability, and collapse. Physical Review Letters. 2006;96(10):104302.

2. Cañizo J, Carrillo J, Rosado J. Collective behavior of animals: Swarming and complex patterns. Arbor. 2010;186:1035–1049.

3. Katz Y, Tunstrøm K, Ioannou CC, Huepe C, Couzin ID. Inferring the structure and dynamics of interactions in schooling fish. Proceedings of The National Academy of Sciences. 2011;108(46):18720–18725.

4. Herbert-Read JE, Perna A, Mann RP, Schaerf TM, Sumpter DJ, Ward AJW. Inferring the rules of interaction of shoaling fish. Proceedings of The National Academy of Sciences. 2011;108(46):18726–18731.

5. Schaerf TM, Dillingham PW, Ward AJW. The effects of external cues on individual and collective behavior of shoaling fish. Science Advances. 2017;3(6):e1603201.

6. Schaerf TM, Herbert-Read JE, Ward AJW. Identifying significant differences in the rules of interaction between individuals in moving animal groups. submitted. 2020;.

7. Hansen MJ, Schaerf TM, Ward AJW. The effect of hunger on the exploratory behaviour of shoals of mosquitofish *Gambusia holbrooki*. Behaviour. 2015;152(12-13):1659–1677.

8. Hansen MJ, Schaerf TM, Ward AJW. The influence of nutritional state on individual and group movement behaviour in shoals of crimson-spotted rainbowfish (*Melanotaenia duboulayi*). Behavioral ecology and sociobiology. 2015;69(10):1713–1722.

9. Tunstrøm K, Katz Y, Ioannou CC, Huepe C, Lutz MJ, Couzin ID. Collective states, multistability and transitional behavior in schooling fish. PLoS Comput Biol. 2013;9(2):e1002915.
