# Supplementary material for: Examination of an averaging method for estimating repulsion and attraction interactions in moving groups
Source: PLoS One. 2020 Dec 9;15(12):e0243631. doi: 10.1371/journal.pone.0243631 (PMC7725364; doi:10.1371/journal.pone.0243631)
Supplement: Attachment — Submitted filename: PONE-D-20-22104.pdf [file pone.0243631.s007.pdf]

This is a very thorough and detailed article where two models, one a prescriptive list of behavioral rules, the other a system of ODEs is used to determine local, mean changes to the speed and direction of individual particles interacting with others. The paper should be published after some minor revisions/discussions are included. They are as listed below

-On page 9 (around line 215) there are some redundant definitions, like \hat u_i, \hat v_i that have already been introduced earlier, in Equations 2.1 and 2.2. So there should be an effort to consolidate this part of the narrative which has a lot of notation.

-Section 2.5 does not have a title

-There is not a lot of information given on how the emergent behavior arises as a function of initial conditions. Indeed, if the same parameters are kept in both models, with individuals kept far enough from each other, perhaps different configurations that the ones shown in Table 2 and 3 would be observed. Also, what happens at the boundaries? Some discussion on boundary conditions/space is necessary.

-Also, I would presume the number of individuals matters in this case, with more/less individuals giving rise to different configurations.

-Perhaps the authors should consider doing some tests i) doubling the physical space, ii) increasing/decreasing the number of individuals, iii) having open and/or reflecting boundary conditions.

-Table 2 has a lot of information, but is there any overall insight as to when cohesion leads to alignment and when it does not? Also, are there cases where cohesion is never seen and the particles disperse? It seems that cohesion arises almost all the time, so maybe they should try cases where (as mentioned above) the individual particles have a bit more space than the 8 r_a^2 area initially allotted to them (this is said on page 9)

-In Table 3, what is the number of individuals they are considering?

-Interesting work that should be cited/used for comparison:

The papers by Chuang, using the same ODE model, contain some interesting other metrics, such as polarity and angular momentum. Have the authors considered using these quantities to describe and better classify their structures? Relevant papers to be referred to are:

YL Chuang et al, State transitions and the continuum limit for a 2D interacting, self, propelled particle system. Physica D 232 (2007) 33-47;

Also, for completeness there is some work done on immersing these particles in fluid environments, and in 3D. Perhaps the in conclusions the authors should discuss how they expect their results to change in such scenarios. This in relevant especially since the authors refer to fish behavior. Relevant papers to be referred to are:

YL Chuang et al, Swarming in viscous fluids: Three dimensional patterns in swimmer and force induced flows, PRE 93 (2016) 043112

NHP Nguyen et al, Thermal and athermal three-dimensional swarms of self-propelled particles, PRE 86 (2012) 011136

Finally, how would these averaging rules apply to other models? Here are other real-life applications of swarming models that could be discussed to enlighten the work of communities that actually try to infer collective movement rules from data/movement.

(Pelagic Fish)

ABT Barbaro et al, Discrete and continuous models of the dynamics of pelagic fish: application to the capelin, Mathematics and Computers in Simulation 79 (2009) 3397-3414

(Scoters)

R. Lukeman et al, Inferring individual rules from collective behavior, PNAS 107 (2010), 12576-12580

(Locusts)

CM Topaz et al, Locust dynamics: behavioral phase change and swarming, PLoS Comp Bio 8 (2012) e1002642

CM Topaz et al, A model for rolling swarms of locusts, Euro Phys J 157 (2008) 93-109

G Ariel et al, Locust Collective motion and its modeling, PLoS Comp Bio 11 (2015) e1004522
