# Supplementary material for: Examination of an averaging method for estimating repulsion and attraction interactions in moving groups
Source: PLoS One. 2020 Dec 9;15(12):e0243631. doi: 10.1371/journal.pone.0243631 (PMC7725364; doi:10.1371/journal.pone.0243631)
Supplement: Attachment — Submitted filename: ResponsestoReviewerComments 1.pdf [file pone.0243631.s008.pdf]

Dear Dr Kryven,

Thank you very much for handling our manuscript, and thank you as well to both the reviewers who took the time to provide useful and considered feedback on the work.

We've made major revisions to the manuscript based on the comments of both reviewers, and have tried to balance reviewer 2's suggestions for reducing the length of the text, with suggestions for additional calculations from both reviewers. As part of our revisions we have moved sections of text describing data binning, the zonal self-propelled particle model that we used, the analytical results for pairwise interactions and estimation of blind zone angles to a supplementary information document. In addition, we have moved many of the figures that appeared in the main text in our initial submission to the supplementary information, resulting in a total reduction from 31 to 11 figures in the main text. Based on suggestions from both reviewers, we performed additional longer duration calculations using the self-propelled particle model, and applied an algorithmic (rather than visual) approach for classifying emergent states for this additional set of calculations. We have also made some additional investigations of group size effects based on the suggestions of reviewers 1 and 2. Our detailed responses to the reviewers' comments are listed below.

On behalf of myself and Rajnesh Mudaliar, and with kind regards,

Timothy Schaerf.

Editorial comments:

2. Thank you for stating in your Funding Statement:

"This work was supported in part by funding from the Australian Research Council's Discovery Project scheme, under project code DP190100660."

*Please provide an amended statement that declares *all* the funding or sources of support (whether external or internal to your organization) received during this study, as detailed online in our guide for authors at http://journals.plos.org/plosone/s/submit-now. Please also include the statement "There was no additional external funding received for this study." in your updated Funding Statement.*

Please include your amended Funding Statement within your cover letter. We will change the online submission form on your behalf.

Response: We have amended the acknowledgement statement to conclude with:

"This work was supported by funding from the Australian Research Council under project DP190100660. There was no additional external funding received for this study."

(Lines 618 to 620 of the manuscript.)

3. Please include captions for your Supporting Information files at the end of your manuscript, and update any in-text citations to match accordingly. Please see our Supporting Information guidelines for more information: *http://journals.plos.org/plosone/s/supporting-information*.

Response: We have included a caption at the end of the manuscript outlining the Supporting Information files. (Lines 621 to 627.)

Review Comments to the Author

Reviewer #1:

This is a very thorough and detailed article where two models, one a prescriptive list of behavioral rules, the other a system of ODEs is used to determine local, mean changes to the speed and direction of individual particles interacting with others. The paper should be published after some minor revisions/discussions are included. They are as listed below

-On page 9 (around line 215) there are some redundant definitions, like \hat u_i, \hat v_i that have already been introduced earlier, in Equations 2.1 and 2.2. So there should be an effort to consolidate this part of the narrative which has a lot of notation.

Response: This text (part of the description of the zonal simulation model) has now been moved to the supporting information, so this duplication of variable definitions no longer occurs in the main text.

-Section 2.5 does not have a title

Response: This section has now been removed, with the text consolidated with other details of the averaging/force-matching method for inferring interactions. (Lines 178 to 188.)

-There is not a lot of information given on how the emergent behavior arises as a function of initial conditions. Indeed, if the same parameters are kept in both models, with individuals kept far enough from each other, perhaps different configurations that the ones shown in Table 2 and 3 would be observed.

Response: We intended the focus of this work to be the ability of the averaging method to correctly (or approximately) extract underlying model prescribed interactions from trajectory data, rather than to be a detailed study of the relationship between initial conditions and emergent behaviour, so we have not delved into this further in our

revisions for this paper. This could however be a good idea for future work aimed at making the relationship between initial conditions and emergent behaviour more explicit; perhaps this could be done more rigorously by examining emergent states as a function of, or in relation to, the order parameters for polarisation and angular momentum that you suggested further below? We have now referenced relevant past work on emergent states in the paper however, in particular the number of time steps required for an emergent state to stabilise (Lines 247-249, 310-312), including a note that it is stated in the work by Bhaskar et al. from 2019 (Chaos 29:123125) that the ODE model that we used is not usually dependent on initial conditions (however for larger groups with N = 200), which is actually contrary to what we found with our smaller groups.

Without additional analysis, it is still clear that the emergent states that we observed in our work are dependent on initial conditions for both models though, given that we observed different emergent states for different initial conditions with all other model parameters kept the same (see Tables 2, ST1, and 3).

Also, what happens at the boundaries? Some discussion on boundary conditions/space is necessary.

Response: All calculations for both models were performed over an unbounded domain (in two spatial dimensions). We've included text in Section 3.1 (line 213) and Section 3.4 (lines 309-310) explicitly noting the unbounded domain.

-Also, I would presume the number of individuals matters in this case, with more/less individuals giving rise to different configurations.

Response: In response to this comment, and a comment by reviewer 2, we have performed some additional calculations for groups of different sizes (although these are not as comprehensive as the core calculations for the study) (Lines 113, 240-242, 304-306, 386-389, Figure 6, 416-412, 533-534, 538-539, SI Section S7, Fig SF22, SI Section S11, Fig SF42-SF51). We did find differences in the emergent states for differing group sizes (see for example, Figures 6 and SF22), although this is not emphasised in the manuscript.

-Perhaps the authors should consider doing some tests i) doubling the physical space, ii) increasing/decreasing the number of individuals, iii) having open and/or reflecting boundary conditions.

Response: Thank you for all these suggestions. In response: (i) As the domains for our simulations were unbounded, it's not really meaningful to double the physical space. (ii) We performed additional calculations that involved increasing and decreasing the number of individuals, with results illustrated in Figures 6, SF22, and SF42-SF51 (in the Supporting Information). (iii) We are preparing another paper that explicitly examines the effects of a reflecting boundary on the sort of analysis described in our paper (using data derived from the alignment interaction only model of Vicsek et al. from 1995).

-Table 2 has a lot of information, but is there any overall insight as to when cohesion leads to alignment and when it does not? Also, are there cases where cohesion is never seen and the particles disperse? It seems that cohesion arises almost all the time, so maybe they should try cases where (as mentioned above) the individual particles have a bit more space than the 8 r_a^2 area initially allotted to them (this is said on page 9)

Response: We did not examine the transition from cohesion to alignment explicitly in our revisions, nor the effects of the size of the initial area occupied by simulated individuals, (again because the intended focus of the work was on assessing the averaging method), but this is a potential avenue to pursue for future work. However, we did observe group fragmentation when simulations were run over longer durations (as suggested by reviewer 2; see for example Figure SF21), and when group size was modified (in response to comments about the potential effects of group size from both reviewers; see for example Figure SF22).

-In Table 3, what is the number of individuals they are considering?

Response: The core calculations using the ODE model were for groups of N = 10 individuals. We've now included this information in the caption for Table 3 (second line of Table 3 caption).

-Interesting work that should be cited/used for comparison:

The papers by Chuang, using the same ODE model, contain some interesting other metrics, such as polarity and angular momentum. Have the authors considered using these quantities to describe and better classify their structures? Relevant papers to be referred to are:

YL Chuang et al, State transitions and the continuum limit for a 2D interacting, self, propelled particle system. Physica D 232 (2007) 33-47;

Response: Thank you for this suggestion. Based on this suggestion we applied a more algorithmic approach to classifying emergent states for the new longer duration simulations using the zonal model, based around calculation of your suggested order parameters of polarisation and angular momentum (as applied by Tunstrøm et al. (2013), PLoS Computational Biology **9**(2):e1002915), and group fragmentation (based on an algorithm for identifying subgroups described in Hansen et al. (2015), Behaviour 152(12-13):16172-16177) (Lines 260-273 (main text), Section S4 of the SI, and Table ST1). Given the importance of the model described by D'Orsogna et al. (2006) to our study, we have referenced the above related paper by Chuang et al. among work on modelling collective motion in our introduction as well.

Also, for completeness there is some work done on immersing these particles in fluid environments, and in 3D. Perhaps the in conclusions the authors should discuss how they expect their results to change in such scenarios. This in relevant especially since the authors refer to fish behavior. Relevant papers to be referred to are:

YL Chuang et al, Swarming in viscous fluids: Three dimensional patterns in swimmer and force induced flows, PRE 93 (2016) 043112

NHP Nguyen et al, Thermal and athermal three-dimensional swarms of self-propelled particles, PRE 86 (2012) 011136

Response: Based on the comments that follow regarding applications to other models, we now note in the introduction that the approach we've applied for validating an averaging/force-matching method could be adapted to analysis in three dimensions, using data from a number of models that work in three dimensions, including the above reference by Chuang et al. (Lines 102-104)

Finally, how would these averaging rules apply to other models? Here are other real-life applications of swarming models that could be discussed to enlighten the work of communities that actually try to infer collective movement rules from data/movement.

Response: We now note in the introduction that "The approach used to examine the averaging method here is quite general, and could have been applied using data generated by any individual based model for collective motion where the positions of individuals are tracked explicitly, irrespective of the species modelled." In addition, we have now referenced the paper by Lukeman et al. (below) as one of the sequence of papers that led to the explicit inference of interaction rules from trajectory data (Lines 75 to 76; the work by Lukeman et al. was in fact very influential on the analysis presented in the work by Herbert-Read et al. from 2011, and should not have been omitted from our original submission).

(Pelagic Fish)

ABT Barbaro et al, Discrete and continuous models of the dynamics of pelagic fish: application to the capelin, Mathematics and Computers in Simulation 79 (2009) 3397-3414

(Scoters)

R. Lukeman et al, Inferring individual rules from collective behavior, PNAS 107 (2010), 12576-12580

(Locusts)

CM Topaz et al, Locust dynamics: behavioral phase change and swarming, PLoS Comp Bio 8 (2012) e1002642

CM Topaz et al, A model for rolling swarms of locusts, Euro Phys J 157 (2008) 93-109

G Ariel et al, Locust Collective motion and its modeling, PLoS Comp Bio 11 (2015) e1004522

Reviewer #2: *The current article investigates the resolution and accuracy of force maps as a tool to understand collective behaviour. To properly benchmark this common tool, the authors have used 2 theoretical models of collective behaviour, and tried to extract some of the properties of the model, such as regions of repulsion, attraction and alignment.*

While I have some harsh criticism in the form the authors chose to present this manuscript, they did present a valid and meaningful scientific study, even if it requires major revisions.

The manuscript was written in a "text book" or "thesis" format, where the authors defined and explained every single equation and procedure used in their paper, most of them multiple times! Even without a page limit, and knowing that papers should be written to a naive audience, the authors took this to the extreme. As a review, thesis or a class project, this paper is great, but as a publication, a very small proportion of people will be willing to read 8 pages (mostly of repetitive definitions) before even properly describing the models they used. The authors even describe their definition of the data binning. In short, the first 18 pages of the paper would normally have been summarised in 3 or 4 pages at most. Similarly, the number of figures in this paper feels excessive, normally most of them would be presented as SI material.

Response: Based on these comments we have moved large portions of the text from the initial submission to a supplementary information (SI) document. The sections that have been moved to the SI include the process for data binning (noted above), the description of the zonal model, the analytical results for pairwise interactions, and the equation used in estimating blind angles. In addition, the bulk of the figures that were in the main text for our initial submission have been moved to the SI document. We have retained the description of the averaging method in the main text, because the focus of the work is to see how good (or how flawed) that form of method is, irrespective of the source of the validation data.

My second criticism of the paper comes in the quantity and duration of simulations the authors have used. 1000 time steps and 10 simulations for a zone model with 25 agents? I understand the authors have explored a large region of the parameter space, and that their results might not change significantly if they expand their stats, but with 1000 time steps one cannot even be sure the results are not a pure result of the initial conditions, and with only 10 simulations this is just unacceptable. I would suggest a minimum of 10^5 time steps, where the first half is discarded, and 100 or more simulations per condition. Similarly for the ODE model, increasing the length of each simulation by an order of magnitude, while noting that the number of simulations is never described in the text (but only in table 3).

Response: Our initial thinking in devising this study was to use data sets that were somewhat comparable in size to those used in experimental studies from which rules of interaction were inferred. For example, in Schaerf et al. 2017, experimental treatments were divided into ten replicates, each of which provided 9000 time steps of data for 8 individuals. Given such data, can the rules of interaction be properly inferred using an averaging method? Based on this rationale to try to match the amount of data that is provided for an experimental study, however, 1000 time steps does seem short.

Therefore, based on the above comment, we repeated the full analysis of the zonal model with 25 individuals, with the duration of all simulations extended to 10000 time steps (Main text: Lines 242-249, 260-273, 277-282, 325-329, 352-385, Fig.5, 601-602, SI Section S6).

Based on reviewer 1's comments, we applied a more algorithmic method for classifying the emergent states for these revised calculations (see section S4 of the SI). In addition, we performed a sequence of calculations to examine the potential effects of initial conditions/transition to stable emergent pattern on our analysis, by separately applying the averaging technique to the first and second halves of a subset of our 10000 time step calculations (SI Section S8). There is little change in the overall results when comparing analysis of 1000 and 10000 time step calculations (Fig. 5), except for a deeper emphasis of an unusual effect trailing the focal individual for parallel groups (bottom row of Fig. 5), and the most striking differences when comparing the first and second halves of 10000 time step calculations relate to fewer bins being covered by data from the latter half of the calculations (Figs. SF23-SF25). Thus the averaging techniques seem to produce similar results for limited or increased duration data sets, and the qualitative form of the inferred interactions seem very similar, even if data is cut to account for transient effects. We also explored analysis of 100000 time step calculations (as suggested above), but such analysis was not possible on our current server, nor tractable in a reasonable amount of time (we had to apply for an extension of time for our revisions to complete the analysis of the 10000 time step zonal model data set). However, we now note that with the initial systematic analysis of the model that was reported in Couzin et al. 2002, 5000 time steps is stated as sufficient duration for an emergent state to stabilise, albeit for simulations of 100, rather than 25, individuals (Lines 247-249), and Bhaskar et al. 2019 note that approximately 2000 time steps (up to t = 100 with dt = 0.05) are sufficient to reach stability using the ODE model (lines 310-312).

The number of replicates per parameter set (10 to 80) and the duration of simulations (10000 time steps) now more closely resemble the amount of data that would be produced by an experimental study, which was our original intention, so thank you for making these suggestions.

With respect to the number of simulations we have amended the sentence in the main text to read "Ten simulations were performed for each combination of zone sizes and associated emergent states; we chose this number of replicates because it is comparable to the number of experimental replicates performed per treatment in experimental

studies that have used the averaging method [35].” (Lines 252-254.) In addition, with respect to the duration of simulations, we have written “The latter, 10000 time step duration simulations have a similar length (in terms of time steps) to the data used in some empirical studies, such as [35], which had 9000 time steps of data per experimental trial for groups of 8 individuals.” (Lines 244-247.) In addition, we have noted in the introduction “Here, we seek to use data of similar duration, and representative of similar numbers of individuals to that derived from experimental studies where averaging methods have been used as part of the analysis.” (Lines 96-99.)

A third criticism (but this is optional), was not to explore the effect of population size in the results, it felt like a missed opportunity.

Response: We performed additional calculations to examine group size effects based on data from both models based on this comment. (Lines 113, 240-242, 304-306, 386-389, Figure 6, 416-412, 533-534, 538-539, SI Section S7, Fig SF22, SI Section S11, Fig SF42-SF51).

One last possible issue comes from a publication [1] that came probably after the authors had submitted this paper. Considering the necessity of major revisions, I feel that the authors should also compare their work to this new one, in addition to Heras et al.
[1] Escobedo R., Lecheval V., Papaspyros V., Bonnet F., Mondada F., Sire C. and Theraulaz G. 2020A data-driven method for reconstructing and modelling social interactions in moving animal groupsPhil. Trans. R. Soc. B37520190380http://doi.org/10.1098/rstb.2019.0380

Response: We have included reference to the above paper in our revised manuscript, in the context of: a method for inferring interactions connected to burst and coast dynamics (along with the earlier work of Calovi et al.) (Line 82); one of the suspected drawbacks of the averaging/force-matching posited in Escobedo et al. being borne out by our analysis (a lack of data relating to repulsion interactions in the analysis of groups of 5, as simulated by the ODE model; lines 417-412, SI Figures SF42 and SF47); and the potential adaptation of some of the approach used by Escobedo et al. to treat turning responses as a sum of repulsion, orientation and attraction effects (Lines 595-598).

In general the work by Escobedo et al. is quite critical of the use of the averaging approach (force maps/force-matching) for inferring interactions, but that element of their work is not supported by the breadth or depth of calculations that we’ve tried to incorporate into this study.

Smaller issues:

Figure 2 makes it clear that the radius of the regions are much larger than the maximum turning rate, meaning most of the interactions are given by the max value of the model.

How do these values compare to the original Couzin publication? If they are similar, this could be a criticism of the model. Also, figure 2 has no units in the color label.

Response: The original Couzin et al. 2002 examination of the zonal model constrained turns at a rate of 40 degrees per second, with simulation time steps of 0.1 second. We used the same settings for our work, and it is true that our figure (now figure SF1) makes it clear that the turning constraint is quite strong, and reaches its maximum in response to partners that are just 4 degrees (or more) out of line with the axis of motion of a focal individual. I (TMS) would not go so far to make this as a criticism of the model though; on paper it seems like a reasonable choice for keeping a parameter constant to allow more focus on how the sizes of interaction zones might affect emergent behaviour.

Lastly, the colors of the heatmaps are in general not colorblind friendly. While not essential, the final range of colors was just distracting, Figure 3 being the most extreme example.

Response: We're very sorry about this. I (TMS) have used the same colour scale for a few different studies, and did not think about how the images might appear to someone that is colour-blind, nor have any previous reviewers mentioned this issue. Is the main issue to do with distinguishing between red and green regions on the plots? If that is the case, then we can re-render the plots using a different colour scale that does not include both red and green, but we wanted to ask for some more information before redoing all our plots.

Thank you again to both reviewers.
